# Blast exposure and long-term diagnoses among veterans: a millennium cohort study investigation of high-level blast and low-level blast

**DOI:** 10.3389/fneur.2025.1599351

**Published:** 2025-07-31

**Authors:** Sarah L. Martindale, Claire A. Kolaja, Jennifer N. Belding, Lynne Liu, Rudolph P. Rull, Daniel W. Trone, Jared A. Rowland

**Affiliations:** ^1^Salisbury VA Healthcare System, Salisbury, NC, United States; ^2^Veterans Integrated Service Network (VISN)-6 Mid-Atlantic Mental Illness, Research Education and Clinical Center (MIRECC), Durham, NC, United States; ^3^Wake Forest School of Medicine, Winston-Salem, NC, United States; ^4^Naval Health Research Center, San Diego, CA, United States; ^5^Leidos, Inc., San Diego, CA, United States; ^6^VA Puget Sound Health Care System, Seattle, WA, United States

**Keywords:** blast, blast induced neurotrauma, low level blast, military, veteran, veteran affairs, psychiatric diagnoses, traumatic brain injury

## Abstract

**Background:**

The effects of blast overpressure (BOP) on brain health are increasingly recognized, yet longitudinal research into these effects after separation from military service remains limited. This study assessed the association between high-level blast (HLB) and low-level blast (LLB) exposure during military service using data from the Millennium Cohort Study (MCS) and diagnoses related to traumatic brain injury (TBI) diagnosed in the Veterans Health Administration (VHA).

**Method:**

MCS participants were included in the analytic sample if they responded to the 2013 survey, were separated from military service, and utilized VHA care for at least 2 years. HLB exposure was assessed using self-report of injury from a “blast/explosion/bullet” in the 2013 survey; LLB risk was determined using military occupational specialty as a proxy. Clinical diagnoses of five TBI severity levels (e.g., mild, penetrating), 22 TBI-related conditions (e.g., tinnitus, dementia/delirium, fatigue) and 10 mental health conditions (e.g., adjustment, bipolar, schizophrenia) were identified using ICD diagnosis codes. Modified Poisson regression with robust error variance was used to examine the relationships between HLB, LLB, and their interaction, adjusting for demographic and military characteristics for each diagnosis of interest.

**Results:**

Statistically significant associations were found between HLB and several TBI diagnoses, TBI-related conditions, and mental health conditions. LLB exposure was associated with only one TBI condition, eight TBI-related conditions, and two mental health conditions. In addition, significant interactions between HLB and LLB were observed for two TBI-related conditions and four mental health conditions.

**Conclusion:**

This study contributes to the growing body of evidence on the long-term effects of BOP on brain health. These findings may inform policy development and educational resources, provide metrics to calculate the potential financial burden on the VHA and increase understanding of long-term health outcomes associated with blast exposure. By utilizing a prospective design and examining VHA diagnoses, the research highlights the potential enduring effects of blast exposure that may continue to require healthcare services after military separation.

## Introduction

Many military service members experience exposures during service that may adversely affect their long-term health (e.g., physical injuries, mental health conditions) (1, 2). Comprehensive healthcare during service is provided by the Military Health System (MHS) whereas the Veterans Health Administration (VHA) provides to eligible veterans following separation. To better inform healthcare service requirements across the lifespan, it is important to understand how military experiences affect service members’ long-term health and subsequent demand for care provided by the VHA. Without adequate healthcare, veterans may experience difficulty managing long-term conditions, which may lead to diminished quality of life and long-term disability (3–6).

Blast overpressure (BOP) is an exposure that warrants further attention. Several literature reviews on health outcomes associated with BOP have been published (7–11). Previous research supports that BOP exposure can impact brain health (8, 12, 13). Specifically, BOP exposure has been associated with many long-term brain health outcomes including increased reporting of psychological symptoms (14), psychiatric diagnoses (15), poorer cognitive function (16, 17), as well as ensuing differences in brain structure (18–21), altered brain function (22, 23), and biomarkers of injury (24) observed years after BOP exposure.

Measuring blast overpressure (BOP) exposure in the military environment is inherently complex due to substantial variability in magnitude, frequency, and context of exposure (e.g., closed location, protective equipment use). Although there are many dimensions for characterizing BOP exposures, one important consideration is whether BOP was the result of incoming or outgoing munitions (25). High-level blast (HLB) refers to BOP from incoming munitions such as improvised explosive devices, whereas the term low-level blast (LLB) refers to BOP from outgoing munitions such as shoulder-fired weapons or breaching charges (25). Notably, these definitions are conceptual rather than strictly based on measurement of peak BOP values. Despite differences in BOP exposure levels and definitions across studies (26), evidence supports the conclusion that both HLB and LLB exposures are associated with adverse health effects, although the long-term health consequences that may emerge following separation from military service has not been sufficiently studied to date.

High-level blast exposure is the leading cause of traumatic brain injury (TBI) among deployed service members (27), contributing to many TBI-related outcomes such as prolonged symptoms and delayed recovery (1, 28–30). For example, prior work observed that HLB-related mild TBI (i.e., concussion) was associated with an increased number of post-concussive symptoms (31) that persist longer than those following impact-related mild TBI (32). Additionally, there is growing evidence suggesting that repetitive HLB exposure may have a cumulative impact, exacerbating both cognitive and emotional symptoms over time (33). Furthermore, cardiovascular and metabolic disorders have also been linked to HLB, suggesting that health effects of HLB extend beyond the brain and impact overall physical health (34).

A recent epidemiological study evaluating health records of over 2.2 million service members found that military occupations with a high risk of LLB exposure had a significantly increased likelihood of being diagnosed with TBI, postconcussive symptoms, or a behavioral health condition (1). Additionally, studies of service members participating in training scenarios (e.g., breachers, heavy weapons training) have shown acute and chronic effects on cognitive function (35–37), postconcussive symptoms (38), brain function (33), neuroinflammation (39), and DNA methylation patterns (40), even in the absence of a documented TBI diagnosis. Furthermore, previous research has noted that service members who had clinical diagnoses of concussion and associated conditions were more likely to be medically separated from service when they also worked in occupations at high risk for LLB (41).

Most examinations of the individual and combined effects of HLB and LLB exposure on humans focus solely on health outcomes that manifest during service. Additionally, relatively few prior investigations had prospective designs. One previous analysis from the U.S. Millennium Cohort Study (MCS), the largest and longest-running prospective study of the health of service members and veterans, sought to address these limitations by examining associations among single and repeated HLB and LLB, respectively on self-reported diagnoses of illness and injury (15). BOP was associated with greater risk of self-report of several clinical diagnoses (e.g., PTSD, hearing loss, migraines). However, this analysis was limited to self-reported diagnoses rather than diagnoses documented in medical records and did not distinguish whether participants received these diagnoses during or following military service. The present research expands this earlier work by estimating effects of HLB and LLB on diagnoses of TBI, TBI-related conditions, and mental health conditions diagnosed in the VHA following separation from service.

## Method

### Participants

Data were from the MCS (42–44). Enrollment methods have been previously described (45, 46). Briefly, service members were randomly selected from Defense Manpower Data Center (DMDC) rosters and invited to enroll in five panels in 2001–2003, 2004–2006, 2007–2008, 2011–2013 and 2020–2021 with over 260,000 participants enrolled to date. Follow-up surveys with questions covering physical and mental health, health behaviors, and life experiences were sent to enrolled participants every 3–5 years (43). Throughout this manuscript, we refer to the survey cycles by the year they closed (e.g., 2013 for the 2011–2013 cycle).

**Figure 1:**
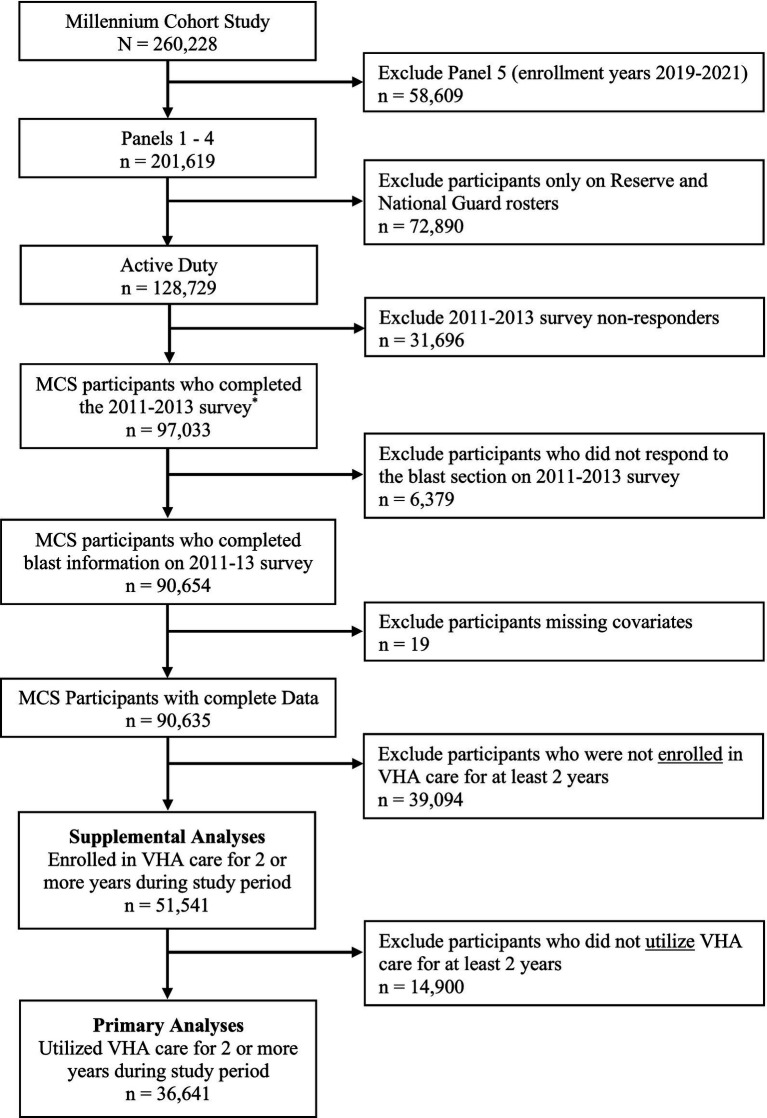
Flow chart for primary and supplemental analytic samples. ^*^The study sample includes all participants who completed the 2011–2013 survey, regardless of whether the survey was a follow-up or baseline for the panel.

Figure 1 depicts the flow chart of inclusion criteria for the present research. MCS participants from Panels 1–4 who served on active duty and completed the 2013 survey, either as baseline or follow-up, were eligible for inclusion (*n* = 97,033). Participants were excluded if responses to the blast screening questions assessed on the 2013 survey (*n* = 6,379) or covariates of interest (*n* = 19) were missing. Because the primary focus of the present analyses was on medical diagnoses recorded in the VHA system, the present analyses were restricted to veterans who utilized VHA care (i.e., were seen for at least one healthcare visit for two or more calendar years; *n* = 36,641). Supplemental analyses were also conducted for the broader population of veterans enrolled in the VHA regardless of the number of visits over time (*n* = 51,541). Individual analyses for each condition of interest were further limited by excluding participants diagnosed with the condition in the VHA prior to the 2013 survey, which resulted in varying analytic sample sizes for each outcome of interest.

### Measures

#### Primary exposures

Self-reported HLB exposure was assessed from the 2013 MCS survey consistent with previously used methods (15). Specifically, participants reported whether they had experienced an injury from a blast, explosion, or bullet and those who endorsed any such injury reported the number of such injuries. Whereas prior research categorized participants into three groups (no HLB, single HLB, or repeated HLB) (15), the present analysis combined single and repeated HLB into one group due to the small number of VHA utilizers who met inclusion criteria. LLB exposure was assessed using DMDC records of military occupation specialty (MOS) codes as a proxy; participants were categorized into high- vs. low-risk of exposure to LLB (1, 15, 41). This approach has been used in previous studies with this MCS population (15) and is supported by a validation study demonstrating that MOS-based risk categories correspond closely with clinically-evaluated LLB exposure (47). Although not a direct measure of BOP, using MOS allows for large-scale estimation of occupational risk when objective exposure data are unavailable.

#### Covariates

Participant characteristics such as sex, race, ethnicity, and birth year were obtained from DMDC at study enrollment. Sex, race, and ethnicity were self-reported to the Department of Defense (DoD) at time of accession into the military. Time-varying military factors such as paygrade and service branch represented status at the time of completion of the 2013 survey. Total time deployed in days was calculated based on dates in and out of theater as reported in the Contingency Tracking System (CTS). Additionally, participants were categorized as not deployed, deployed without combat, or deployed with combat based on CTS data and self-reported combat experiences reported on any MCS survey through the 2013 survey (48). Marital status and education attainment were self-reported on the 2013 survey and backfilled with DMDC information if missing. Study panel was included as a covariate in adjusted models to adjust for potential heterogeneity.

#### Outcomes of interest

Conditions of interest were identified in VHA inpatient and outpatient records during the study period (i.e., completion of the 2013 survey through the 2021 fiscal year). The MHS Data Repository (MDR) was used to identify cases diagnosed before the 2013 survey and outside of VHA. Broadly, diagnoses of interest fell into three categories: traumatic brain injury (i.e., any TBI, mild TBI, moderate TBI, severe TBI, penetrating TBI), TBI-related conditions (e.g., conditions commonly comorbid with concussion), and mental health conditions (e.g., posttraumatic stress disorder [PTSD], adjustment disorder). Conditions were identified using case criteria established by the Armed Forces Health Surveillance Division (AFHSD) or prior literature [e.g., (1, 49, 50)]. Whereas prior research evaluated associations between blast exposure and clinical diagnoses of substance abuse, the present research did not because substance use-related conditions diagnosed within the VHA are not currently available due to federal protections from Title 38 of U. S. Code § 7,332. Supplementary Table 1 lists case criteria and diagnoses.

### Data analysis

Descriptive statistics were calculated for military and demographic characteristics overall and stratified by occupational risk of LLB and self-reported HLB exposure. The geometric mean and standard deviation were calculated for the number of days deployed because this continuous variable displayed a log-normal distribution (51). Collinearity among covariates was examined with a variance inflation factor threshold of 4. For each condition, we calculated the numbers of cases diagnosed: (a) overall [i.e., in either the MDR or VHA medical systems], (b) in the MDR before the 2013 survey, (c) in the VHA before the 2013 survey, and (d) in the VHA after the 2013 survey. Although counts and frequencies are presented for all four criteria, this report will focus on cases identified in the VHA during the study period. Adjusted models were calculated excluding participants diagnosed in the VHA before the 2013 survey. Prevalence ratios for HLB exposure and LLB occupation for each condition were estimated using Poisson regression in the GENMOD procedure with the robust standard errors (52). Models adjusted for military (service branch, total days deployed, pay grade, deployment/combat experience) and demographic factors (age, sex, race, ethnicity, education, marital status), enrollment panel, diagnosis of the condition in the MDR before the 2013 survey, and the interaction between HLB and LLB. When models failed to converge due to a small number of participants with a prior diagnosis of the condition in the MDR before the 2013 survey (i.e., for memory loss and delirium/dementia), cases with prior history were excluded from adjusted analyses. False discovery rate (FDR) adjustment for multiple comparisons was used to identify significant direct associations and interaction effects (53). FDR corrections were chosen instead of traditional family-wise error rate corrections as they confer increased statistical power, which was deemed appropriate considering the smaller sample size than similar published investigations (15). When the two-way interaction variable between HLB and LLB was statistically significant (FDR corrected *p* < 0.05), adjusted associations were calculated with a common reference category of blast-naïve (i.e., those with no HLB and low risk of LLB exposure). Although we did not set *a priori* criteria for number of cases to conduct analyses, some models failed to converge, therefore adjusted prevalence ratios were not calculated. All analyses were conducted in SAS 9.4.

## Results

### Descriptive

Table 1 reports sample characteristics for the full sample and by occupational risk for LLB and self-reported HLB exposure. Among the analytic sample of veterans who utilized VHA care for two or more years during the study period (*N* = 36,641), most participants were enrolled in the 2013 survey, male, identified as White/non-Hispanic race and ethnicity, were born after 1979, attended some college but had not obtained a degree, were married, were enlisted, and served in the Army (Table 1). The average total time deployed was 302 days (geometric *SD* = 2.49). Approximately 17.4% of the sample worked in an occupation at high risk for LLB exposure; the most common high-risk occupations included general infantry (37.9%), combat operations control (12.6%), and combat engineering (8.1%). In addition, 10.6% of the sample reported HLB exposure on the 2013 MCS survey. All military and demographic characteristics examined were significantly different by occupational risk of LLB exposure and self-reported HLB exposure.

**Table 1:** Demographic and military characteristics among veterans enrolled in the millennium cohort study who utilized VHA for 2 or more years.

Characteristic	All *n* = 36,641		Low-level blast (LLB)				High-level blast (HLB)			
			No *n* = 30,257		Yes *n* = 6,384		No *n* = 32,752		Yes *n* = 3,889	
	*n*	(%)	*n*	(%)	*n*	(%)	*n*	(%)	*n*	(%)
Panel (Enrollment years)										
1 (2001–2003)	12,071	(32.9)	10,459	(34.6)	1,612	(25.3)	11,100	(33.9)	971	(25.0)
2 (2004–2006)	4,419	(12.1)	3,625	(12.0)	794	(12.4)	3,841	(11.7)	578	(14.9)
3 (2007–2008)	6,597	(18.0)	5,494	(18.2)	1,103	(17.3)	5,787	(17.7)	810	(20.8)
4 (2011–2013)	13,554	(37.0)	10,679	(35.3)	2,875	(45.0)	12,024	(36.7)	1,530	(39.3)
Sex										
Male	25,108	(68.5)	19,274	(63.7)	5,834	(91.4)	21,633	(66.1)	3,475	(89.4)
Female	11,533	(31.5)	10,983	(36.3)	550	(8.6)	11,119	(33.9)	414	(10.6)
Race and ethnicity										
American Indian	595	(1.6)	472	(1.6)	123	(1.9)	520	(1.6)	75	(1.9)
Asian or Pacific Islander	1,524	(4.2)	1,306	(4.3)	218	(3.4)	1,412	(4.3)	112	(2.9)
Black, non-Hispanic	5,055	(13.8)	4,512	(14.9)	543	(8.5)	4,752	(14.5)	303	(7.8)
White, non-Hispanic	25,714	(70.2)	20,851	(68.9)	4,863	(76.2)	22,655	(69.2)	3,059	(78.7)
Hispanic	3,260	(8.9)	2,692	(8.9)	568	(8.9)	2,954	(9.0)	306	(7.9)
Multiracial	493	(1.3)	424	(1.4)	69	(1.1)	459	(1.4)	34	(0.9)
Birth year										
Before 1970	7,565	(20.6)	6,708	(22.2)	857	(13.4)	6,989	(21.3)	576	(14.8)
1970–1979	8,488	(23.2)	7,092	(23.4)	1,396	(21.9)	7,572	(23.1)	916	(23.6)
After 1979	20,588	(56.2)	16,457	(54.4)	4,131	(64.7)	18,191	(55.5)	2,397	(61.6)
Education										
High School Diploma/equivalent or less	5,027	(13.7)	3,524	(11.6)	1,503	(23.5)	4,269	(13.0)	758	(19.5)
Some college, no degree	15,338	(41.9)	12,215	(40.4)	3,123	(48.9)	13,494	(41.2)	1,844	(47.4)
Associates degree	5,612	(15.3)	4,759	(15.7)	853	(13.4)	5,110	(15.6)	502	(12.9)
Bachelors degree	6,439	(17.6)	5,732	(18.9)	707	(11.1)	5,919	(18.1)	520	(13.4)
Masters or higher	4,225	(11.5)	4,027	(13.3)	198	(3.1)	3,960	(12.1)	265	(6.8)
Marital status										
Single, never married	8,002	(21.8)	6,550	(21.6)	1,452	(22.7)	7,316	(22.3)	686	(17.6)
Now married	22,059	(60.2)	18,143	(60.0)	3,916	(61.3)	19,581	(59.8)	2,478	(63.7)
No longer married	6,580	(18.0)	5,564	(18.4)	1,016	(15.9)	5,855	(17.9)	725	(18.6)
Pay grade										
Enlisted	31,384	(85.7)	25,101	(83.0)	6,283	(98.4)	27,861	(85.1)	3,523	(90.6)
Officer	5,257	(14.3)	5,156	(17.0)	101	(1.6)	4,891	(14.9)	366	(9.4)
Service branch										
Army	16,543	(45.1)	12,656	(41.8)	3,887	(60.9)	13,736	(41.9)	2,807	(72.2)
Navy/Coast Gard	6,653	(18.2)	5,702	(18.8)	951	(14.9)	6,472	(19.8)	181	(4.7)
Marine Corps	4,604	(12.6)	3,539	(11.7)	1,065	(16.7)	3,935	(12.0)	669	(17.2)
Air Force	8,841	(24.1)	8,360	(27.6)	481	(7.5)	8,609	(26.3)	232	(6.0)
Days deployed (geometric mean, std. dev)	301.66 (2.49)		280.94 (2.56)		397.89 (2.03)		283.97 (2.53)		438.57 (1.94)	
Deployment experience										
Not deployed	11,609	(31.7)	10,414	(34.4)	1,195	(18.7)	11,305	(34.5)	304	(7.8)
Deployed, without combat	3,626	(9.9)	3,298	(10.9)	328	(5.1)	3,585	(10.9)	41	(1.1)
Deployed, with combat	21,406	(58.4)	16,545	(54.7)	4,861	(76.1)	17,862	(54.5)	3,544	(91.1)
High-level blast										
No	32,752	(89.4)	28,131	(93.0)	4,621	(72.4)	32,752	(100.0)	<30	(<1.0)
Yes	3,889	(10.6)	2,126	(7.0)	1,763	(27.6)	0	(0)	3,889	(100.0)
Low-level blast										
No	30,257	(82.6)	30,257	(100.0)	0	(0)	28,131	(85.9)	2,126	(54.7)
Yes	6,384	(17.4)	0	(0)	6,384	(100.0)	4,621	(14.1)	1,763	(45.3)
High-risk LLB occupations										
Armor and Amphibious, General					391	(6.1)	277	(6.0)	114	(6.5)
Artillery and Gunnery					485	(7.6)	360	(7.8)	125	(7.1)
Aviation Ordnance					511	(8.0)	492	(10.7)	< 30	(< 1.0)
Combat Engineering, General					514	(8.1)	327	(7.1)	187	(10.6)
Combat Operations Control, General					807	(12.6)	561	(12.1)	246	(14.0)
EOD/UDT					193	(3.0)	143	(3.1)	50	(2.8)
Expeditionary Medical Service					270	(4.2)	226	(4.9)	44	(2.5)
Infantry, General					2,419	(37.9)	1,522	(32.9)	897	(50.9)
Infantry, Gun Crews, and Seamen					173	(2.7)	173	(3.7)	< 30	(< 1.0)
Military Training Instructor					148	(2.3)	142	(3.1)	< 30	(< 1.0)
Missile Artillery, Operating					304	(4.8)	227	(6.0)	< 30	(< 1.0)
Rocket Artillery					74	(1.2)	56	(1.2)	< 30	(< 1.0)
Special Forces					95	(1.5)	65	(1.4)	30	(1.7)

*LLB–low-level blast, determined by occupational risk; HLB–high-level blast, self-reported; EOD/UDT–Explosive Ordnance Disposal/Underwater Demolition Team. Sample sizes < 30 have been masked consistent with reporting requirements.

### TBI diagnoses

#### Descriptive

Among the TBI diagnoses recorded in the VHA after the 2013 survey, mild TBI was the most common (3.0%), followed by moderate TBI (2.1%; Table 2). Severe and penetrating TBIs were rare (*n* < 10) and thus not examined further.

**Table 2:** Case counts for traumatic brain injury (TBI) and mental health conditions of interest.

Condition	Utilized VHA care for 2 or more years (n = 36,641)			Diagnosed in VHA after 2013 survey* n (%)		Analytic sample
	Total diagnosed* n (%)	Diagnosed in MDR before 2013 survey* n (%)	Diagnosed in VHA before 2013 survey* n (%)			
TBI diagnoses						
Any TBI^a^	5,778 (15.8)	2,989 (8.2)	843 (2.3)	1,593	4.5	35,798
Mild TBI^a^	5,111 (13.9)	2,780 (7.6)	621 (1.7)	1,069	3.0	36,019
Moderate TBI^a^	1990 (5.4)	675 (1.8)	311 (0.8)	774	2.1	36,330
Severe TBI^a^	79 (0.2)	45 (0.1)	17 (0.0)	5	0.0	36,624
Penetrating TBI^a^	84 (0.2)	62 (0.2)	8 (0.0)	9	0.0	36,633
TBI-related conditions						
Tinnitus^a^	12,147 (33.2)	2,841 (7.8)	2,389 (6.5)	6,632	19.4	34,252
Significant hearing loss^e^	12,112 (33.1)	5,016 (13.7)	2,736 (7.5)	5,764	17.0	33,904
Hearing problems^b^	14,007 (38.2)	4,982 (13.6)	3,189 (8.7)	7,059	21.1	33,452
Dizziness/Vertigo^b^	5,869 (16.0)	891 (2.4)	124 (0.3)	3,138	8.6	36,517
Chronic fatigue syndrome^e^	1,269 (3.5)	0 (0)	0 (0)	857	2.3	36,641
Fatigue^c^	14,354 (39.2)	5,274 (14.4)	552 (1.5)	5,645	15.6	36,087
Sleep apnea^e^	18,589 (50.7)	5,580 (15.2)	2,128 (5.8)	12,236	35.5	34,511
Sleep disorders and symptoms^b^	25,547 (69.7)	10,937 (29.8)	3,423 (9.3)	16,717	50.3	33,213
Sleep disruption movement disorders^d^	1910 (5.2)	349 (1.0)	85 (0.2)	920	2.5	36,556
Gait and coordination problems^b^	2,124 (5.8)	0 (0)	0 (0)	1,477	4.0	36,641
Skin sensation disturbances^b^	6,226 (17.0)	0 (0)	0 (0)	3,915	10.7	36,641
Vision problems^b^	4,360 (11.9)	2,363 (6.4)	294 (0.8)	993	2.7	36,346
Headache^b^	17,104 (46.7)	5,952 (16.2)	1818 (5.0)	11,196	32.2	34,823
Migraine headaches^e^	11,977 (32.7)	4,912 (13.4)	1739 (4.7)	7,454	21.4	34,902
Non-headache pain^b^	34,905 (95.3)	28,576 (78.0)	8,299 (22.6)	21,202	74.8	28,335
Syncope and collapse^b^	5,221 (14.2)	2,495 (6.8)	236 (0.6)	1,598	4.4	36,405
Altered mental status^b^	2,202 (6.0)	602 (1.6)	58 (0.2)	850	2.3	36,583
Cognitive problems^b^	5,863 (16.0)	1,204 (3.3)	431 (1.2)	3,081	8.5	36,210
Communication disorders^b^	829 (2.3)	110 (0.3)	46 (0.1)	376	1.0	36,595
Delirium/Dementia^b^	654 (1.8)	1 (0)	3 (0)	383	1.1	36,638
Memory loss^d^	874 (2.4)	1 (0.0)	283 (0.8)	588	1.6	36,358
Post-concussive syndrome^c^	1,450 (4.0)	765 (2.1)	163 (0.4)	289	0.8	36,641
Mental health diagnoses						
Acute stress disorder^a^	2,439 (6.7)	1,543 (4.2)	163 (0.4)	335	0.9	36,478
ADD/ADHD^b^	3,893 (10.6)	1,105 (3.0)	228 (0.6)	2,502	6.9	36,413
Adjustment disorders^a^	16,651 (45.4)	8,081 (22.1)	3,386 (9.2)	6,667	20.0	33,255
Anxiety disorders^a^	17,365 (47.4)	4,260 (11.6)	1735 (4.7)	11,778	33.7	34,904
Manic-depressive disorder^e^	4,488 (12.2)	1,020 (2.8)	865 (2.4)	2,497	7.0	35,776
Bipolar disorders^a^	2,587 (7.1)	529 (1.4)	393 (1.1)	1,607	4.4	36,248
Depressive disorders^a^	19,416 (53.0)	6,218 (17.0)	3,303 (9.0)	13,093	39.3	33,335
Personality disorders^a^	2068 (5.6)	836 (2.3)	231 (0.6)	916	2.5	36,410
PTSD^a^	14,516 (39.6)	2,450 (6.7)	2,637 (7.2)	10,844	31.9	34,004
Schizophrenia^a^	357 (1.0)	51 (0.1)	75 (0.2)	226	0.6	36,566

^*^Total diagnosed, diagnosed in MDR before 2013 survey, and diagnosed in VHA before 2013 survey are among the entire eligible sample (n = 36,641); Numbers diagnosed in VHA after 2013 survey (outcomes of interest) were calculated among the condition specific analytic sample that excludes cases diagnosed in the VHA before the 2013 survey. ^a^Armed Forces Health Surveillance Division; all conditions required 1 inpatient or 2 outpatient visits within 180 days except for schizophrenia which required 1 inpatient or 4 outpatient visits without a time limit in accordance with these criteria. ^b^Farmer et al. (50); sensitive criteria. ^c^Belding et al. (32); sensitive criteria. ^d^Belding et al. (67); criterion of two inpatient or outpatient visits within 1 year. ^e^Carey et al. (49); sensitive criteria.

#### Adjusted models

In the adjusted models (Table 3), HLB exposure was associated with any TBI, mild TBI, and moderate TBI. Notably, the largest magnitude of association (three-fold increase) between HLB exposure and TBI diagnoses was for moderate TBI. LLB was associated with moderate TBI, but not mild TBI. Similar to HLB, LLB was most strongly associated with moderate TBI diagnosis. There were no significant interaction effects observed between LLB and HLB for TBI diagnoses.

**Table 3:** Adjusted prevalence ratios between HLB and LLB on subsequent mental health, traumatic brain injury and sensory/symptomology diagnosis in the VHA, among millennium cohort study participants who utilized the VHA for 2 or more years.

Condition	Adjusted LLB			Adjusted HLB			HLB x LLB Interaction		
	PR	95% CI	*p*-value*	PR	95% CI	*p*-value*	PR	95% CI	*p*-value*
Traumatic brain injury diagnoses									
Any TBI^a^	1.16	(0.99, 1.35)	0.08	2.69	(2.33, 3.10)	<0.0001	0.87	(0.71, 1.07)	0.19
Mild TBI^a^	1.13	(0.94, 1.36)	0.28	2.61	(2.18, 3.12)	<0.0001	0.97	(0.75, 1.25)	0.80
Moderate TBI^a^	1.33	(1.07, 1.66)	0.03	3.43	(1.79, 4.20)	<0.0001	0.75	(0.56, 1.01)	0.09
Severe TBI^a^	–			–					–
Penetrating TBI^a^	–			–					–
TBI-related conditions									
Tinnitus^a^	1.11	(1.04, 1.18)	0.004	1.27	(1.17, 1.37)	<0.0001	0.98	(0.86, 1.10)	0.69
Significant hearing loss^e^	1.10	(1.03, 1.18)	0.01	1.37	(1.26, 1.48)	<0.0001	0.94	(0.83, 1.07)	0.39
Hearing problems^b^	1.11	(1.04, 1.18)	0.002	1.27	(1.18, 1.37)	<0.0001	0.95	(0.85, 1.06)	0.38
Dizziness/Vertigo^b^	0.96	(0.85, 1.07)	0.49	1.30	(1.14, 1.49)	0.002	1.06	(0.86, 1.32)	0.57
Chronic fatigue syndrome^e^	0.91	(0.72, 1.14)	0.58	0.96	(0.71, 1.31)	0.87	0.85	(0.50, 1.44)	0.64
Fatigue^c^	0.95	(0.88, 1.03)	0.29	1.16	(1.05, 1.28)	0.01	1.01	(0.86, 1.19)	0.93
Sleep apnea^e^	0.97	(0.93, 1.01)	0.10	1.10	(1.05, 1.16)	0.001	0.91	(0.84, 0.99)	0.04
Sleep disorders and symptoms^b^	0.99	(0.96, 1.03)	0.70	1.13	(1.08, 1.17)	<0.0001	0.92	(0.86, 0.97)	0.01
Sleep disruption movement disorders^d^	1.34	(1.10, 1.62)	0.02	1.31	(1.02, 1.69)	0.12	0.96	(0.65, 1.41)	0.88
Gait and coordination problems^b^	1.11	(0.95, 1.30)	0.26	1.45	(1.20, 1.76)	0.003	0.82	(0.59, 1.13)	0.26
Skin sensation disturbances^b^	1.00	(0.91, 1.11)	0.93	1.27	(1.13, 1.42)	0.0008	0.83	(0.68, 1.01)	0.07
Vision problems^b^	1.10	(0.91, 1.34)	0.37	1.47	(1.15, 1.87)	0.02	0.94	(0.64, 1.38)	0.75
Headache^b^	1.07	(1.02, 1.12)	0.01	1.33	(1.26, 1.41)	<0.0001	0.97	(0.89, 1.06)	0.49
Migraine headaches^e^	1.07	(1.00, 1.14)	0.07	1.44	(1.34, 1.55)	<0.0001	1.08	(0.97, 1.21)	0.18
Non-headache pain^b^	1.01	(0.99, 1.03)	0.33	1.08	(1.05, 1.10)	<0.0001	0.97	(0.93, 1.01)	0.12
Syncope and collapse^b^	1.07	(0.92, 1.24)	0.43	1.48	(1.23, 1.78)	0.002	0.75	(0.55, 1.01)	0.10
Altered mental status^b^	1.06	(0.87, 1.30)	0.56	1.79	(1.41, 2.25)	0.0002	0.78	(0.54, 1.13)	0.26
Cognitive problems^b^	1.17	(1.06, 1.30)	0.006	1.98	(1.78, 2.21)	<0.0001	0.86	(0.74, 1.01)	0.08
Communication disorders^b^	1.50	(1.12, 2.01)	0.05	1.56	(1.05, 2.31)	0.10	0.79	(0.44, 1.40)	0.49
Delirium/Dementia^b^	1.16	(0.83, 1.61)	0.50	1.94	(1.37, 2.76)	0.02	1.41	(0.84, 2.35)	0.16
Memory loss^d^	1.46	(1.15, 1.85)	0.02	2.63	(2.05, 3.38)	<0.0001	0.81	(0.56, 1.17)	0.38
Post-concussive syndrome^c^	1.13	(0.77, 1.66)	0.70	2.24	(1.54, 3.27)	0.01	1.21	(0.71, 2.09)	0.67
Mental health diagnoses									
Acute stress disorder^a^	1.27	(0.91, 1.78)	0.27	1.88	(1.25, 2.81)	0.10	0.51	(0.26, 1.02)	0.15
ADD/ADHD^b^	1.01	(0.90, 1.13)	0.85	1.02	(0.87, 1.18)	0.85	0.19	(0.95, 1.49)	0.18
Adjustment disorders^a^	1.25	(1.18, 1.33)	<0.0001	1.69	(1.58, 1.81)	<0.0001	0.77	(0.70, 0.86)	<0.0001
Anxiety disorders^a^	1.00	(0.95, 1.04)	0.93	1.09	(1.03, 1.15)	0.001	0.92	(0.84, 1.01)	0.10
Bipolar disorders^a^	1.03	(0.89, 1.20)	0.67	1.40	(1.17, 1.69)	0.002	0.68	(0.50, 0.92)	0.02
Manic-depressive disorder^e^	0.99	(0.88, 1.11)	0.85	1.32	(1.14, 1.52)	0.001	0.87	(0.69, 1.09)	0.24
Depressive disorders^a^	1.03	(0.99, 1.07)	0.25	1.20	(1.15, 1.27)	<0.0001	0.89	(0.83, 0.97)	0.01
Personality disorders^a^	1.12	(0.92, 1.37)	0.36	0.93	(0.69, 1.25)	0.63	1.24	(0.80, 1.92)	0.38
PTSD^a^	1.17	(1.13, 1.23)	<0.0001	1.52	(1.45, 1.59)	<0.0001	0.82	(0.76, 0.87)	<0.0001
Schizophrenia^a^	1.14	(0.79, 1.64)	0.58	1.37	(0.83, 2.27)	0.38	0.80	(0.37, 1.76)	0.58

LLB–low-level blast, determined by occupational risk; HLB–high-level blast, self-reported. Models adjusted for military (service branch, total days deployed, pay grade) and demographic factors (age, sex, race, ethnicity, education, marital status), enrollment panel, diagnosis of the condition in the MDR before the 2013 survey, and the interaction between HLB and LLB. ^*^False discovery rate (FDR) adjusted *p*-values. ^a^Armed Forces Health Surveillance Division; all conditions required 1 inpatient or 2 outpatient visits within 180 days except for schizophrenia was required 1 inpatient or 4 outpatient visits without a time limit in accordance with these criteria. ^b^Farmer et al. (50); sensitive criteria. ^c^Belding et al. (32); sensitive criteria. ^d^Belding et al. (67); criterion of two inpatient or outpatient visits within 1 year. ^e^Carey et al. (49); sensitive criteria.

### TBI-related conditions

#### Descriptive

Among the 22 TBI-related conditions, the five most prevalent were non-headache pain (74.8%), sleep disorders and symptoms (50.3%), sleep apnea (35.5%), headache (32.2%), and migraine headaches (21.4%).

#### Adjusted models

HLB was significantly associated with most conditions in the fully adjusted models except for chronic fatigue syndrome, sleep disruption movement and communication disorders (Table 3). The largest prevalence ratios were seen for memory loss, post-concussive syndrome, cognitive problems, and delirium/dementia. LLB was associated with subsequent tinnitus, significant hearing loss, hearing problems, sleep disruption movement disorders, headache, cognitive problems, memory loss, and communication disorders. The largest prevalence ratios among significant associations were for communication disorders, memory loss, and sleep disruption/movement disorders. Among these conditions, significant HLB and LLB interactions were observed for sleep apnea as well as sleep disorders and symptoms (FDR adjusted *p* = 0.04 and 0.01, respectively). These interactions were antagonistic, such that the combined effects were less than the additive effects. Specifically, when compared with blast-naïve veterans, those who only endorsed HLB exposure were significantly more likely to be diagnosed with sleep apnea or sleep disorders and symptoms in the VHA whereas those who were in a LLB occupation (solely or in combination with HLB exposure) did not appear to be more likely to have a diagnosis (Table 4).

**Table 4:** Adjusted prevalence ratios examining the combined effect between HLB and LLB exposure on subsequent mental health, traumatic brain injury and sensory/symptomology diagnosis in the VHA.

Condition	Ref: Neither	LLB Only	HLB Only	HLB and LLB
TBI-related conditions				
Sleep apnea^c^	Ref	0.97 (0.93, 1.01)	**1.10 (1.05, 1.16)**	0.97 (0.92, 1.03)
Sleep disorders and symptoms^b^	Ref	0.99 (0.96, 1.03)	**1.13 (1.08, 1.17)**	1.03 (0.98, 1.07)
Mental health diagnoses				
Adjustment disorders^a^	Ref	**1.25 (1.18, 1.33)**	**1.69 (1.58, 1.81)**	**1.64 (1.52, 1.76)**
Bipolar disorders^a^	Ref	1.03 (0.89, 1.20)	**1.40 (1.17, 1.69)**	0.98 (0.77, 1.25)
Depressive disorders^a^	Ref	1.03 (0.99, 1.07)	**1.20 (1.15, 1.27)**	**1.10 (1.04, 1.17)**
PTSD^a^	Ref	**1.17 (1.13, 1.23)**	**1.52 (1.45, 1.59)**	**1.46 (1.39, 1.53)**

LLB–low-level blast, determined by occupational risk; HLB–high-level blast, self-reported. Bold typeface indicates *p* < 0.05. Models adjusted for military (service branch, total days deployed, pay grade) and demographic factors (age, sex, race, ethnicity, education, marital status), enrollment panel, diagnosis of the condition in the MDR before the 2013 survey, and the interaction between HLB and LLB. ^a^Armed Forces Health Surveillance Division; all conditions required 1 inpatient or 2 outpatient visits within 180 days. ^b^Farmer et al. (50); sensitive criteria. ^c^Carey et al. (49); sensitive criteria.

### Mental health conditions

#### Descriptive

Among the 10 mental health diagnoses, the most prevalent conditions during the study period were depression (39.3%), anxiety (33.7%), PTSD (31.9%), adjustment disorder (20.0%), and manic-depressive disorder (7.0%).

#### Adjusted models

HLB was significantly associated with adjustment, anxiety, bipolar, manic-depressive, depressive, and PTSD, whereas LLB exposure was associated with adjustment disorder and PTSD (Table 3). The strongest associations between HLB and mental health conditions were seen for acute stress disorder, adjustment disorders, PTSD, bipolar disorders, and manic-depressive disorder. LLB was most strongly associated with acute stress disorder, adjustment disorders, and PTSD.

Interaction effects between HLB and LLB were statistically significant for: adjustment disorder, bipolar disorder, depressive disorders, and PTSD (Table 4). For adjustment disorder and PTSD, compared to blast-naïve veterans, those with blast exposure were more likely to be diagnosed in the VHA. Whereas LLB was independently associated with increased likelihood of diagnoses of these conditions in the VHA, overlapping confidence intervals for HLB only and both HLB and LLB suggest that LLB did not increase the likelihood of PTSD and adjustment disorder diagnosis above and beyond HLB exposure. Similar to TBI-related conditions, these interactions were antagonistic, such that, when compared to blast-naïve veterans, veterans were more likely to be diagnosed with depressive disorders if they reported HLB exposure either alone or with LLB, whereas bipolar disorders were more likely to be diagnosed by those who only experienced HLB (Table 4).

### Supplemental analyses

Supplementary Tables 2–4 display results among veterans enrolled for two or more years in the VHA during the study period (*N* = 51,541), regardless of utilization. Previous reported findings included only VHA utilizers (i.e., those enrolled in the VHA with at least one medical encounter per year for 2 years). Case counts mirrored those observed in the main analyses although rates of conditions were generally lower, consistent with the larger sample size that includes VHA nonusers. Adjusted model results are consistent with a few exceptions (Supplementary Tables 3, 4). Among TBI-related conditions, HLB was additionally associated with sleep disruption movement disorders and communication disorders, whereas LLB was additionally associated with migraine headaches and non-headache pain. The interaction between HLB and LLB was significant for four additional conditions: skin sensation disturbances, non-headache pain, syncope and collapse, and cognitive problems. Like the primary analyses, these interaction effects were antagonistic. Among mental health diagnoses, HLB was associated with acute stress disorder and LLB was additionally associated with depressive disorders.

## Discussion

The present analysis leveraged data from the MCS to estimate associations and interactions between HLB and LLB exposure and diagnoses of TBI, TBI-related conditions, and mental health conditions among veterans who utilize the VHA (i.e., those with at least one medical encounter per year for at least 2 years). These findings add further evidence to the accumulating body of research suggesting that BOP, including both HLB and LLB, increases the occurrence of these outcomes and are among the first to document these associations in active VHA users.

Both HLB and LLB were associated with increased prevalence of TBI. Specifically, HLB was associated with greater likelihood of receiving a diagnosis in the VHA for TBI (i.e., any TBI, moderate TBI), TBI-related conditions (e.g., tinnitus, fatigue, post-concussive syndrome, hearing loss, sleep apnea, headache, non-headache pain, cognitive problems, vision problems), and mental health conditions (i.e., adjustment disorders, anxiety disorders, bipolar disorders, depressive disorders, and PTSD). LLB was similarly associated with greater prevalence of many, although not all, of these diagnoses. Several of the conditions examined could be categorized as, or are related to, post-concussive symptoms. HLB was associated with all conditions investigated apart from chronic fatigue syndrome, sleep disruption movement, and communication disorders. Our findings for headaches and cognitive problems were consistent with research documenting an association between blast exposure and performance on cognitive tasks (17, 35, 36, 54). Prior work has also directly compared performance on neurocognitive testing, often reporting subtle differences in performance. The current results suggest the effect of BOP on cognitive function, even if subtle, may represent a subclinical concern that affects ongoing care provided by the VHA. Additionally, the observed association between LLB and hearing-related conditions underscores the hazard of exposure to loud noises during training activities and subsequent long-term effects on hearing. This adds to a prior finding that LLB was associated with hearing problems in active-duty service members (2) by documenting that hearing problems persist beyond active duty service and continue to require clinical management; tinnitus and hearing loss are the most common service-related veterans disability claims (55).

The effect of LLB alone remained significant when adjusting for interaction effects, suggesting that LLB imparts a unique effect on likelihood of PTSD and adjustment disorders independent of HLB exposure. These diagnoses were identified when veterans received clinical care provided by the VHA, but it remains unclear whether these diagnoses reflect continued treatment for a previous TBI or a subsequent TBI. This raises the possibility of important differences between blast-induced TBI and non-blast TBI regarding recovery from the effects of the injury. Although this has been previously reported from cross-sectional studies (16, 56), the current study provides additional support using longitudinal and medical record data.

Significant interaction effects between HLB and LLB exposure were observed for several TBI-related and mental health conditions, but not for TBI itself. For mental health conditions, individuals with HLB with or without LLB were at increased risk of diagnosis. In contrast, for sleep apnea and other sleep disorders and symptoms, individuals with HLB exposure only were significantly more likely to be diagnosed in the absence of LLB. LLB was not associated with increased likelihood of diagnosis for sleep conditions but was associated with increased risk of adjustment disorder and PTSD both with and without associated HLB.

These findings highlight the need to consider both the independent and interactive effects of different blast exposures when evaluating long-term risks to brain health. For example, HLB and LLB could be associated with distinct underlying pathophysiological mechanisms, such as acute neurotrauma from HLB compared to chronic subclinical inflammatory changes associated with LLB. Further, LLB risk was based on MOS categories, a strategy that could result in groups of individuals with different experiences in other domains such as combat, training, and exposure to trauma in addition to differences in LLB. From this perspective, the interaction effects provide insight into the effects of HLB on these different groups of individuals, suggesting the possibility that the long-term effects of HLB may differ across these groups. For clinical outcomes, the presence of HLB exposure may be a more robust predictor of psychological outcomes than certain neuropsychiatric and sleep-related outcomes. LLB may contribute more subtly, or in the case of MOS, be additionally influenced by other variables such as combat exposure or training, or in ways that are not readily captured. Understanding these differential effects can help inform exposure tracking protocols, guide individualized screening strategies, and refine clinical decision-making for veterans with varying blast exposure histories. Ultimately, this work underscores the complexity of blast-related brain health outcomes and the importance of tailoring interventions to the type and context of exposure.

### Implications for care

Although previous research has documented associations among HLB and LLB on long-term health outcomes, there is a paucity of work evaluating health care utilization within the VHA due to BOP exposure. These results underscore the strong association between blast exposure and adverse health outcomes, particularly the high magnitude of risk observed for TBI, memory loss, and post-concussive syndrome. Veterans exposed to blasts during their military service may continue to face significant repercussions linked to BOP exposure. The conditions examined herein (e.g., TBI, mental health conditions) represent significant burden on health care systems both for the direct clinical management of the condition and the secondary effects on physical health (57, 58). These results highlight that the long-term effects of BOP exposure on physical health and mental health represents a concern not only for the DoD, but also for entities such as VHA that provide care for veterans.

Regarding military health policy and preventive care, our results highlight the need for systematic tracking and planned analysis of both HLB and LLB exposures over the course of a service member’s career. Incorporating blast exposure histories—using tools such as MOS risk categories or standardized self-report instruments—into routine medical records could help the DoD and VHA better anticipate long-term healthcare needs, enable early screening for at-risk individuals, and guide the development of targeted rehabilitation and clinical monitoring protocols. Further, incorporating blast exposure into disability evaluations for VHA should be considered in future. From a policy standpoint, this study supports the implementation of occupational blast exposure surveillance systems and the development of evidence-based exposure thresholds that inform safe training practices, consistent with recent DoD policy (59).

Understanding the full implications for treatment and management of these conditions requires a better understanding of the potential mechanisms (e.g., neurological, psychological) by which BOP exposure may increase likelihood of diagnosis for the observed conditions. Possible effects of BOP exposure on brain structure and function have been previously documented (12, 13, 19, 26, 39, 60, 61). Previous research has noted the association between TBI and psychological health, though it remains unclear whether this is due to structural or functional damage to the brain or psychological mechanisms due to a traumatic event (62).

Of particular concern for preventive efforts are the outcomes of LLB including PTSD, adjustment disorder, hearing conditions, and cognitive problems. These associations were independent of HLB, suggesting the possibility that service members chronically exposed to LLB during training activities may have an increased likelihood of these disorders in the future. This is further supported by findings that chronic LLB exposure, rather than acute LLB exposure, is typically associated with increased symptom reporting (33, 35, 38, 39).

### Strengths, limitations, and future directions

The presented study results need to be considered within the context of limitations. First, HLB was self-reported. Service members reported whether they had been exposed to a blast/explosion (i.e., HLB), but were not asked to provide additional information regarding characteristics of that HLB exposure (e.g., distance, intensity, frequency). Additionally, it was not possible to directly link medical record data to the individually reported HLB events, which limits the ability to verify the severity of specific exposures.

Low-level blast risk was estimated using MOS as a proxy for exposure risk. This method is consistent with prior work (2, 15, 47, 63) and allows for large-scale estimation, but it does not capture individual variation in cumulative exposure, time in service, or specific training environments. However, prior work supports the validity of MOS as a proxy, showing that MOS-based classifications correspond to actual exposure experiences (47). Although imperfect, this method provides a reasonable and validated strategy for modeling LLB exposure in large cohort studies, including the MCS (1, 15). In addition to its utility in epidemiological research, MOS may also hold practical value for identifying individuals at elevated risk for blast-related brain health outcomes. Recently directed tracking of BOP (59) incorporates MOS-based risk stratification into military and post-service healthcare systems which is intended to support targeted mitigation strategies during training (e.g., modified exposure limits, protective equipment), and early identification of individuals who may benefit from clinical monitoring or intervention. This approach is expected to enhance prevention and treatment efforts by allowing the MHS and VHA to proactively address the needs of service members and Veterans most likely to exhibit blast-related health concerns. The continued improvement of objective tools to measure exposure, such as body-mounted sensors, biometric wearables, and biospecimen data, may improve the precision of BOP exposure estimates. Although practical limitations currently restrict the broad application of these tools, they have strong potential to complement self-report and administrative data in future research.

Second, our primary analytic sample focused on veterans who had been identified as VHA users (i.e., enrolled in the VHA with at least one medical encounter per year for at least 2 years), meaning all participants had to meet eligibility criteria that typically require documented health concerns related to military service. Although this sample may be representative of VHA users broadly, generalizability to veterans who do not regularly use or are not eligible for VHA services may be limited (64–66). In addition, there is potential for collider bias, as exposures to blast and experience of conditions may result in a greater likelihood of veterans using VHA care. Veterans from lower-risk occupational backgrounds would therefore likely only be represented in this sample if they exhibited sufficient service-related symptoms to access care, which may bias the sample toward more symptomatic individuals in this group. Regardless, our results provide a valuable source of information regarding cost burden to the government for continued healthcare services following separation from service.

Finally, the analyses from the present research were limited by features of the study design. For example, we were unable to evaluate whether TBI may have moderated the associations between HLB and LLB and other subsequent health outcomes. Detailed TBI data were not available in the 2013 survey cycle, therefore we were unable to determine if TBI diagnoses occurred concurrent to reported HLB. Additionally, not all VHA medical records included diagnosis order, therefore we were unable to apply exact AFHSB case definitions that require the ICD code to be the first or second diagnostic code.

Despite these limitations, the present research has notable strengths including its use of a large, representative sample and prospective design. We were also able to account for several demographic and military factors, including deployment with and without combat experience. These strengths, combined with the ability to link self-report data with VHA medical records, enabled one of the first prospective examinations of long-term health outcomes associated with BOP exposure to date.

## Conclusion

Our findings underscore the significant impact of blast exposure on post-service health outcomes among veterans, including TBI, TBI-related physical health conditions, and mental health diagnoses. These findings add to a growing body of literature about the potential adverse health outcomes associated with blast exposure experienced during military service. We employed a prospective design and ascertained clinical diagnoses recorded in the VHA. These results have potential to inform preventive measures, intervention strategies, and healthcare policies tailored to mitigate the adverse consequences of blast exposure on the well-being of military personnel post-separation. Increased understanding of the effects of HLB and LLB on long-term health outcomes can guide targeted treatment and support mechanisms for individuals exposed to blast during their military service.

The implications of this work extend beyond the U.S. military. International forces employ similar weapon systems and conduct comparable training activities that can generate meaningful levels of overpressure, including breaching, artillery, and shoulder-fired weapons. As such, our findings may inform global efforts to characterize, monitor, and mitigate the effects of BOP exposure across coalition partners. In addition, certain law enforcement agencies, particularly tactical units such as law enforcement Special Weapons and Tactics (SWAT) teams, explosive breachers, and sniper teams, may be subject to similar patterns of LLB exposure during training and operations. These communities may likewise benefit from policy changes aimed at improving exposure tracking, implementing protective measures, and monitoring long-term neurological and psychological outcomes associated with occupational blast exposure. Moving forward, continued research efforts and targeted interventions are essential to mitigate the long-term effects of blast exposure and improve outcomes for military and paramilitary populations.

## Data Availability

The data analyzed in this study is subject to the following licenses/restrictions: Millennium Cohort Study data are not publicly available due to Department of Defense (DoD) and Veterans Affairs (VA) regulations. However, access may be requested through the Millennium Cohort Study team (https://millenniumcohort.org/) for approved research purposes. Additionally, statistical code and detailed analysis steps are available upon reasonable request to facilitate reproducibility. By following these rigorous methodological and reporting standards, this research enhances the credibility, reliability, and reproducibility of findings, contributing to the growing body of knowledge on the long-term health consequences of blast exposure in military populations. Requests to access these datasets should be directed to https://millenniumcohort.org/.

## Author disclaimer

The views, opinions and/or findings contained in this article are those of the authors and should not be construed as an official US Department of Veterans Affairs or US Department of Defense position, policy or decision, unless so designated by other official documentation. SM, JB, LL, RR, DT, and JR are employees of the U. S. Government. This work was prepared as part of our official duties. Title 17, U.S.C. §105 provides that copyright protection under this title is not available for any work of the U.S. Government. Title 17, U.S.C. §101 defines a U.S. Government work as work prepared by a military service member or employee of the U.S. Government as part of that person’s official duties. The views expressed in this article are those of the authors and do not necessarily reflect the official policy or position of the Department of the Navy, Department of Defense, Department of Veterans Affairs, nor the U. S. Government. The study protocol was approved by the Naval Health Research Center Institutional Review Board in compliance with all applicable Federal regulations governing the protection of human subjects. Research data were derived from an approved Naval Health Research Center Institutional Review Board protocol, number NHRC.2000.0007 and VA Puget Sound Institutional Review Board project #1587777.

## References

[1] Belding JN Englert R Bonkowski J Thomsen CJ. Occupational risk of low-level blast exposure and TBI-related medical diagnoses: a population-based epidemiological investigation (2005–2015). Int J Environ Res Public Health. (2021) 18:24. doi: 10.3390/ijerph182412925 PMC8700773 34948535

[2] Carr W Kelley AL Toolin CF Weber NS. Association of MOS-based blast exposure with medical outcomes. Front Neurol. (2020) 11:619. doi: 10.3389/fneur.2020.00619, PMID: 32849167 PMC7413071

[3] Baker MS. Casualties of the global war on terror and their future impact on health care and society: a looming public health crisis. Mil Med. (2014) 179:348–55. doi: 10.7205/MILMED-D-13-00471, PMID: 24690957

[4] Dalton MK Jarman MP Manful A Koehlmoos TP Cooper Z Weissman JS. The hidden costs of war: healthcare utilization among individuals sustaining combat-related trauma (2007-2018). Ann Surg. (2023) 277:159–64. doi: 10.1097/SLA.0000000000004844, PMID: 33651722

[5] Geiling J Rosen JM Edwards RD. Medical costs of war in 2035: long-term care challenges for veterans of Iraq and Afghanistan. Mil Med. (2012) 177:1235–44. doi: 10.7205/MILMED-D-12-00031, PMID: 23198496

[6] Hale-Gallardo J Jia H Delisle T Levy CE Osorio V Smith JA. Enhancing health and independent living for veterans with disabilities by leveraging community-based resources. J Multidiscip Healthc. (2017) 10:41–7. doi: 10.2147/JMDH.S118706, PMID: 28182140 PMC5279827

[7] Belanger HG Bowling F Yao EF. Low-level blast exposure in humans a systematic review of acute and chronic effects. J Spec Oper Med. (2020) 20:87–93. doi: 10.55460/3ac6-ax9i, PMID: 32203612

[8] Belding JN Englert RM Fitzmaurice S Jackson JR Koenig HG Hunter MA. Potential health and performance effects of high-level and low-level blast: a scoping review of two decades of research. Front Neurol. (2021) 12:628782. doi: 10.3389/fneur.2021.628782, PMID: 33776888 PMC7987950

[9] Greer N Sayer N Koeller E Velasquez T Wilt TJ. Outcomes associated with blast versus nonblast-related traumatic brain injury in US military service members and veterans: a systematic review. J Head Trauma Rehabil. (2018) 33:E16–29. doi: 10.1097/HTR.0000000000000304, PMID: 28422897

[10] Ravula AR Das T Gosain A Dolalas T Padhi S Chandra N. An update on repeated blast traumatic brain injury. Curr Opinion Biomed Engin. (2022) 24:100409. doi: 10.1016/j.cobme.2022.100409

[11] Simmons MM Engel CC Hoch E Orr P Anderson B Azhar GS. Neurological effects of repeated exposure to military occupational levels of blast: A review of scientific literature RAND (2020) (Accessed January 15, 2025).

[12] Cernak I. Understanding blast-induced neurotrauma: how far have we come? Concussion. (2017) 2:CNC42. doi: 10.2217/cnc-2017-0006, PMID: 30202583 PMC6093818

[13] Siedhoff HR Chen S Song H Cui J Cernak I Cifu DX. Perspectives on primary blast injury of the brain: translational insights into non-inertial low-intensity blast injury. Front Neurol. (2022) 12:818169. doi: 10.3389/fneur.2021.818169, PMID: 35095749 PMC8794583

[14] Martindale SL Ord AS Rule LG Rowland JA. Effects of blast exposure on psychiatric and health symptoms in combat veterans. J Psychiatr Res. (2021) 143:189–95. doi: 10.1016/j.jpsychires.2021.09.021, PMID: 34500348

[15] Belding JN Kolaja CA Rull RP Trone DW. Single and repeated high-level blast, low-level blast, and new-onset self-reported health conditions in the U.S. millennium cohort study: an exploratory investigation. Front Neurol. (2023) 14:1110717. doi: 10.3389/fneur.2023.1110717, PMID: 37025202 PMC10070873

[16] Bailie J Lippa S Hungerford L French LM Brickell TA Lange RT. Cumulative blast exposure during a military career negatively impacts recovery from traumatic brain injury. J Neurotrauma. (2023). doi: 10.1089/neu.2022.0192 37675903

[17] Martindale SL Ord AS Rowland JA. Influence of blast exposure on cognitive functioning in combat veterans. Neuropsychology. (2020) 34:735–43. doi: 10.1037/neu0000672, PMID: 32673000 PMC8363214

[18] Martindale SL Rowland JA Shura RD Taber KH. Longitudinal changes in neuroimaging and neuropsychiatric status of post-deployment veterans: a CENC pilot study. Brain Inj. (2018) 32:1208–16. doi: 10.1080/02699052.2018.1492741, PMID: 29985673

[19] Martindale SL Shura RD Rostami R Taber KH Rowland JA. Research letter: blast exposure and brain volume. J Head Trauma Rehabil. (2021) 36:424–8. doi: 10.1097/HTR.0000000000000660, PMID: 33656482

[20] Taber KH Hurley RA Haswell CC Rowland JA Hurt SD Lamar CD. White matter compromise in veterans exposed to primary blast forces. J Head Trauma Rehabil. (2015) 30:E15–25. doi: 10.1097/HTR.0000000000000030, PMID: 24590156 PMC4470620

[21] Trotter BB Robinson ME Milberg WP McGlinchey RE Salat DH. Military blast exposure, ageing and white matter integrity. Brain. (2015) 138:2278–92. doi: 10.1093/brain/awv139, PMID: 26033970 PMC4840948

[22] Robinson ME Clark DC Milberg WP McGlinchey RE Salat DH. Characterization of differences in functional connectivity associated with close-range blast exposure. J Neurotrauma. (2017) 34:S-53–61. doi: 10.1089/neu.2016.4709, PMID: 28486051

[23] Robinson ME Lindemer ER Fonda JR Milberg WP McGlinchey RE Salat DH. Close-range blast exposure is associated with altered functional connectivity in veterans independent of concussion symptoms at time of exposure. Hum Brain Mapp. (2015) 36:911–22. doi: 10.1002/hbm.22675, PMID: 25366378 PMC6869346

[24] Edwards KA Greer K Leete J Lai C Devoto C Qu B-X. Neuronally-derived tau is increased in experienced breachers and is associated with neurobehavioral symptoms. Sci Rep. (2021) 11:19527. doi: 10.1038/s41598-021-97913-0, PMID: 34593828 PMC8484560

[25] Belding JN Egnoto M Englert RM Fitzmaurice S Thomsen CJ. Getting on the same page: consolidating terminology to facilitate cross-disciplinary health-related blast research. Front Neurol. (2021) 12:695496. doi: 10.3389/fneur.2021.695496, PMID: 34248831 PMC8264539

[26] Rowland JA Martindale SL. Considerations for the assessment of blast exposure in service members and veterans. Front Neurol. (2024) 15:1383710. doi: 10.3389/fneur.2024.1383710, PMID: 38685944 PMC11056521

[27] Bell RS Vo AH Neal CJ Tigno J Roberts R Mossop C. Military traumatic brain and spinal column injury: a 5-year study of the impact blast and other military grade weaponry on the central nervous system. J Trauma. (2009) 66:S104–11. doi: 10.1097/TA.0b013e31819d88c8, PMID: 19359953

[28] Belding JN Fitzmaurice S Englert RM Lee I Kowitz B Highfill-McRoy RM. Blast exposure and risk of recurrent occupational overpressure exposure predict deployment TBIs. Mil Med. (2020) 185:e538–44. doi: 10.1093/milmed/usz289, PMID: 31665414

[29] DBRIC (2020) FY20 prevention, mitigation, and treatment of blast injuries report to the executive Agena. Available online at: https://blastinjuryresearch.health.mil/assets/docs/ea_report/FY20_Report_to_the_Executive_Agent.pdf (Accessed January 15, 2025).

[30] Rowland JA Martindale SL Spengler KM Shura RD Taber KH. Sequelae of blast events in Iraq and Afghanistan war veterans using the Salisbury blast interview: a CENC study. Brain Inj. (2020) 34:642–52. doi: 10.1080/02699052.2020.1729418, PMID: 32096666 PMC9007162

[31] Belding JN Fitzmaurice S Englert RM Koenig HG Thomsen CJ Olaghere da Silva U. Self-reported concussion symptomology during deployment: differences as a function of injury mechanism and low-level blast exposure. J Neurotrauma. (2020) 37:2219–26. doi: 10.1089/neu.2020.6997, PMID: 32368945

[32] Belding JN Khokhar B Englert RM Fitzmaurice S Thomsen CJ. The persistence of blast- versus impact-induced concussion symptomology following deployment. J Head Trauma Rehabil. (2021) 36:E397–405. doi: 10.1097/HTR.0000000000000715, PMID: 34320556

[33] Stone JR Avants BB Tustison NJ Wassermann EM Gill J Polejaeva E. Functional and structural neuroimaging correlates of repetitive low-level blast exposure in career breachers. J Neurotrauma. (2020) 37:2468–81. doi: 10.1089/neu.2020.7141 32928028 PMC7703399

[34] Finlay SE Earby M Baker DJ Murray VSG. Explosions and human health: the long-term effects of blast injury. Prehosp Disaster Med. (2012) 27:385–91. doi: 10.1017/S1049023X12000891, PMID: 22800859

[35] Carr W Stone JR Walilko T Young LA Snook TL Paggi ME. Repeated low-level blast exposure: a descriptive human subjects study. Mil Med. (2016) 181:28–39. doi: 10.7205/MILMED-D-15-00137, PMID: 27168550

[36] LaValle CR Carr WS Egnoto MJ Misistia AC Salib JE Ramos AN. Neurocognitive performance deficits related to immediate and acute blast overpressure exposure. Front Neurol. (2019) 10:949. doi: 10.3389/fneur.2019.00949, PMID: 31572285 PMC6754066

[37] Vartanian O PhD Coady L MSc Blackler K MSc Fraser B BA Cheung B PhD. Neuropsychological, neurocognitive, vestibular, and neuroimaging correlates of exposure to repetitive low-level blast waves: evidence from four nonoverlapping samples of Canadian breachers. Mil Med. (2021) 186:e393–400. doi: 10.1093/milmed/usaa332, PMID: 33135742

[38] Carr W Polejaeva E Grome A Crandall B LaValle C Eonta SE. Relation of repeated low-level blast exposure with symptomology similar to concussion. J Head Trauma Rehabil. (2015) 30:47–55. doi: 10.1097/HTR.0000000000000064, PMID: 24901327

[39] Stone JR Avants BB Tustison NJ Gill J Wilde EA Neumann KD. Neurological effects of repeated blast exposure in special operations personnel. J Neurotrauma. (2024) 41:neu.2023.0309. doi: 10.1089/neu.2023.0309, PMID: 37950709 PMC11001960

[40] Wang Z Wilson CM Ge Y Nemes J LaValle C Boutté A. DNA methylation patterns of chronic explosive breaching in U.S. military warfighters. Front Neurol. (2020) 11:1010. doi: 10.3389/fneur.2020.01010, PMID: 33192958 PMC7645105

[41] Belding JN Bonkowski J Englert R. Traumatic brain injury and occupational risk of low-level blast exposure on adverse career outcomes: an examination of administrative and medical separations from service (2005–2015). Front Neurol. (2024) 15:1389757. doi: 10.3389/fneur.2024.1389757, PMID: 38689879 PMC11058224

[42] Belding JN Castañeda SF Jacobson IG LeardMann CA Porter B Powell TM. The millennium cohort study: the first 20 years of research dedicated to understanding the long-term health of US service members and veterans. Ann Epidemiol. (2022) 67:61–72. doi: 10.1016/j.annepidem.2021.12.002, PMID: 34906635

[43] Castañeda SF Belding JN Kolaja CA LeardMann CA Jacobson IG Rivera AC. Cohort profile update: the US millennium cohort study—evaluating the impact of military experiences on service members and veteran health. Int J Epidemiol. (2023) 52:e222–31. doi: 10.1093/ije/dyad088, PMID: 37348866

[44] Chesbrough KB Ryan MAK Amoroso P Boyko EJ Gackstetter GD Hooper TI. The millennium cohort study: a 21-year prospective cohort study of 140,000 military personnel. Mil Med. (2002) 167:483–8. doi: 10.1093/milmed/167.6.483, PMID: 12099084

[45] Ryan MAK Smith TC Smith B Amoroso P Boyko EJ Gray GC. Millennium cohort: enrollment begins a 21-year contribution to understanding the impact of military service. J Clin Epidemiol. (2007) 60:181–91. doi: 10.1016/j.jclinepi.2006.05.009, PMID: 17208125

[46] Smith B Smith TC Gray GC Ryan MAK for the Millennium Cohort Study Team. When epidemiology meets the internet: web-based surveys in the millennium cohort study. Am J Epidemiol. (2007) 166:1345–54. doi: 10.1093/aje/kwm212, PMID: 17728269

[47] Martindale SL Belding JN Crawford CD Rowland JA. Validation of military occupational specialty as a proxy for blast exposure using the Salisbury blast interview. J Neurotrauma. (2023) 40:2321–9. doi: 10.1089/neu.2023.0067, PMID: 37058360

[48] Porter B Hoge CW Tobin LE Donoho CJ Castro CA Luxton DD. Measuring aggregated and specific combat exposures: associations between combat exposure measures and posttraumatic stress disorder, depression, and alcohol-related problems. J Trauma Stress. (2018) 31:296–306. doi: 10.1002/jts.22273, PMID: 29603393

[49] Carey FR Harbertson J Sharifian N Boyko EJ Rull RP. All-cause mortality among United States military personnel: findings from the millennium cohort study, 2001–2021. Ann Epidemiol. (2024) 99:1–8. doi: 10.1016/j.annepidem.2024.08.006, PMID: 39214485

[50] Farmer CM Krull H Concannon TW Simmons M Pillemer F Ruder T. Understanding treatment of mild traumatic brain injury in the military health system. Rand Health Q. (2017) 6:11. 10.7249/rhq-06-02-11 PMC5568165 28845349

[51] Benedict TJ Anthony K David AA. Empirical comparison of relative precision of geometric measure of variation about the mean and standard deviation. International Journal of Recent Engineering Research And Development. (2019) 4:1–12. doi: 10.31730/osf.io/tzkw9

[52] Zou G. A modified poisson regression approach to prospective studies with binary data. Am J Epidemiol. (2004) 159:702–6. doi: 10.1093/aje/kwh090, PMID: 15033648

[53] Benjamini Y Hochberg Y. Controlling the false discovery rate: a practical and powerful approach to multiple testing. J Royal Statistic Soc Series B. (1995) 57:289–300. doi: 10.1111/j.2517-6161.1995.tb02031.x

[54] Vartanian O Tenn C Rhind SG Nakashima A Di Battista AP Sergio LE. Blast in context: the neuropsychological and neurocognitive effects of long-term occupational exposure to repeated low-level explosives on Canadian Armed Forces’ breaching instructors and range staff. Front Neurol. (2020) 11:588531. doi: 10.3389/fneur.2020.588531, PMID: 33343492 PMC7744759

[55] Veterans Benefits Administration Annual benefits report fiscal year 2022. (2022). Available online at: https://www.benefits.va.gov/REPORTS/abr/docs/2022-abr.pdf (Accessed January 15, 2025).

[56] Ord AS Epstein EL Shull ER Taber KH Martindale SL Rowland JA. Factors associated with recovery from posttraumatic stress disorder in combat veterans: the role of deployment mild traumatic brain injury (mTBI). Rehabil Psychol. (2022) 67:356–68. doi: 10.1037/rep0000400, PMID: 35420867 PMC9338889

[57] Barnes DE Byers AL Gardner RC Seal KH Boscardin WJ Yaffe K. Association of mild traumatic brain injury with and without loss of consciousness with dementia in US military veterans. JAMA Neurol. (2018) 75:1055–61. doi: 10.1001/jamaneurol.2018.0815, PMID: 29801145 PMC6143113

[58] Izzy S Chen PM Tahir Z Grashow R Radmanesh F Cote DJ. Association of Traumatic Brain Injury with the risk of developing chronic cardiovascular, endocrine, neurological, and psychiatric disorders. JAMA Netw Open. (2022) 5:e229478. doi: 10.1001/jamanetworkopen.2022.9478, PMID: 35482306 PMC9051987

[59] Deputy Secretary of Defense (2024) Department of Defense Requirements for managing brain health risks from blast overpressure (memorandum for senior pentagon leadership Nos. OSD005281-24/CMD007440-24; pp. 1–3). Available online at: https://axonmedicaltech.com/wp-content/uploads/2024/08/DEPARTMENT-OF-DEFENSE-REQUIREMENTS-FOR-MANAGING-BRAIN-HEALTH-RISKS-FROM-BLAST-OVERPRESSURE-OSD005281-24-RES-FINAL.pdf

[60] Li C Chen S Siedhoff HR Grant D Liu P Balderrama A. Low-intensity open-field blast exposure effects on neurovascular unit ultrastructure in mice. Acta Neuropathol Commun. (2023) 11:144. doi: 10.1186/s40478-023-01636-4, PMID: 37674234 PMC10481586

[61] Rutter B Song H DePalma RG Hubler G Cui J Gu Z. Shock wave physics as related to primary non-impact blast-induced traumatic brain injury. Mil Med. (2021) 186:601–9. doi: 10.1093/milmed/usaa290, PMID: 33499439

[62] Rowland JA Stapleton-Kotloski JR Godwin DW Hamilton CA Martindale SL. The functional connectome and long-term symptom presentation associated with mild TBI and blast exposure in combat veterans. J Neurotrauma. (2024). doi: 10.1089/neu.2023.0315 39150013

[63] Belding J. N. Jackson J. R. Englert R. M. Koenig H. Thomsen C. J. (2020). Blast exposure, traumatic brain injury, and self-reported symptomology among active duty enlisted marines: an examination of post-deployment health assessment records, 2005-2012. 227.

[64] Weeks WB West AN Wallace AE Fisher ES. Comparing the characteristics, utilization, efficiency, and outcomes of VA and non-VA inpatient care provided to VA enrollees: a case study in New York. Med Care. (2008) 46:863–71. doi: 10.1097/MLR.0b013e31817d92e1, PMID: 18665066

[65] West AN Charlton ME Vaughan-Sarrazin M. Dual use of VA and non-VA hospitals by veterans with multiple hospitalizations. BMC Health Serv Res. (2015) 15:431. doi: 10.1186/s12913-015-1069-8, PMID: 26416176 PMC4587652

[66] Yoon J Phibbs CS Ong MK Vanneman ME Chow A Redd A. Outcomes of veterans treated in veterans affairs hospitals vs non–veterans affairs hospitals. JAMA Netw Open. (2023) 6:e2345898. doi: 10.1001/jamanetworkopen.2023.45898, PMID: 38039003 PMC10692833

[67] Belding JN Bonkowski J Englert R Grimes Stanfill A Tsao JW. Associations between concussion and more severe TBIs, mild cognitive impairment, and early-onset dementia among military retirees over 40 years. Front Neurol. (2024) 15:1442715. doi: 10.3389/fneur.2024.1442715 39296958 PMC11408918

