# Supplementary material for: Blast exposure and long-term diagnoses among veterans: a millennium cohort study investigation of high-level blast and low-level blast
Source: Front Neurol. 2025 Jul 31;16:1599351. doi: 10.3389/fneur.2025.1599351 (PMC12352330; doi:10.3389/fneur.2025.1599351)
Supplement: Supplementary file 1 [file Table_1.docx]

| **Supplemental Table 1.** Diagnostic codes used for case ascertainment. | |
| --- | --- |
| **Condition** | **Diagnostic Codes** |
| **Traumatic Brain Injury (TBI) Diagnoses** | |
| Mild TBI^a^ | V155_2, V155_7, V155_C, V1552_2, V1552_7, V1552_C, V1559_2, V1559_7, V1559_C, 850, 8500, 8501, 8505, 8509, 85011, 95901 |
| Moderate TBI^a^ | V155_3, V155_8, V155_D, V1552_3, V1552_8, V1552_D, V1559_3, V1559_8, V1559_D, 8502, 80000, 80001, 80002, 80003, 80006, 80009, 80010, 80011, 80012, 80013, 80016, 80019, 80020, 80021, 80022, 80023, 80026, 80029, 80030, 80031, 80032, 80033, 80036, 80039, 80040, 80041, 80042, 80043, 80046, 80049, 80100, 80101, 80102, 80103, 80106, 80109, 80110, 80111, 80112, 80113, 80116, 80119, 80120, 80121, 80122, 80123, 80126, 80129, 80130, 80131, 80132, 80133, 80136, 80139, 80140, 80141, 80142, 80143, 80146, 80149, 80300, 80301, 80302, 80303, 80306, 80309, 80310, 80311, 80312, 80313, 80316, 80319, 80320, 80321, 80322, 80323, 80326, 80329, 80330, 80331, 80332, 80333, 80336, 80339, 80340, 80341, 80342, 80343, 80346, 80349, 80400, 80401, 80402, 80403, 80406, 80409, 80410, 80411, 80412, 80413, 80416, 80419, 80420, 80421, 80422, 80423, 80426, 80429, 80430, 80431, 80432, 80433, 80436, 80439, 80440, 80441, 80442, 80443, 80446, 80449, 85012, 85100, 85101, 85102, 85103, 85106, 85109, 85120, 85121, 85122, 85123, 85126, 85129, 85140, 85141, 85142, 85143, 85146, 85149, 85160, 85161, 85162, 85163, 85166, 85169, 85180, 85181, 85182, 85183, 85186, 85189, 85200, 85201, 85202, 85203, 85206, 85209, 85220, 85221, 85222, 85223, 85226, 85229, 85240, 85241, 85242, 85243, 85246, 85249, 85300, 85301, 85302, 85303, 85306, 85309, 85400, 85401, 85402, 85403, 85406, 85409 |
| Severe TBI^a^ | V155_5, V155_A, V155_F, V1552_5, V1552_A, V1552_F, V1559_5, V1559_A, V1559_F, 80050, 80051, 80052, 80053, 80054, 80055, 80056, 80059, 80060, 80061, 80062, 80063, 80064, 80065, 80066, 80069, 80070, 80071, 80072, 80073, 80074, 80075, 80076, 80079, 80080, 80081, 80082, 80083, 80084, 80085, 80086, 80089, 80090, 80091, 80092, 80093, 80094, 80095, 80096, 80099, 80150, 80151, 80152, 80153, 80154, 80155, 80156, 80159, 80160, 80161, 80162, 80163, 80164, 80165, 80166, 80169, 80170, 80171, 80172, 80173, 80174, 80175, 80176, 80179, 80180, 80181, 80182, 80183, 80184, 80185, 80186, 80189, 80190, 80191, 80192, 80193, 80194, 80195, 80196, 80199, 80350, 80351, 80352, 80353, 80354, 80355, 80356, 80359, 80360, 80361, 80362, 80363, 80364, 80365, 80366, 80369, 80370, 80371, 80372, 80373, 80374, 80375, 80376, 80379, 80380, 80381, 80382, 80383, 80384, 80385, 80386, 80389, 80390, 80391, 80392, 80393, 80394, 80395, 80396, 80399, 80450, 80451, 80452, 80453, 80454, 80455, 80456, 80459, 80460, 80461, 80462, 80463, 80464, 80465, 80466, 80469, 80470, 80471, 80472, 80473, 80474, 80475, 80476, 80479, 80480, 80481, 80482, 80483, 80484, 80485, 80486, 80489, 80490, 80491, 80492, 80493, 80494, 80495, 80496, 80499, 85110, 85111, 85112, 85113, 85114, 85115, 85116, 85119, 85130, 85131, 85132, 85133, 85134, 85135, 85136, 85139, 85150, 85151, 85152, 85153, 85154, 85155, 85156, 85159, 85170, 85171, 85172, 85173, 85174, 85175, 85176, 85179, 85190, 85191, 85192, 85193, 85194, 85195, 85196, 85199, 85210, 85211, 85212, 85213, 85214, 85215, 85216, 85219, 85230, 85231, 85232, 85233, 85234, 85235, 85236, 85239, 85250, 85251, 85252, 85253, 85254, 85255, 85256, 85259, 85310, 85311, 85312, 85313, 85314, 85315, 85316, 85319, 85410, 85411, 85412, 85413, 85414, 85415, 85416, 85419 |
| Penetrating TBI^a^ | V155_4, V155_9, V155_E, V1552_4, V1552_9, V1552_E, V1559_4, V1559_9, V1559_E, 8503, 8504, 80004, 80005, 80014, 80015, 80024, 80025, 80034, 80035, 80044, 80045, 80104, 80105, 80114, 80115, 80124, 80125, 80134, 80135, 80144, 80145, 80304, 80305, 80314, 80315, 80324, 80325, 80334, 80335, 80344, 80345, 80404, 80405, 80414, 80415, 80424, 80425, 80434, 80435, 80444, 80445, 85104, 85105, 85124, 85125, 85144, 85145, 85164, 85165, 85184, 85185, 85204, 85205, 85224, 85225, 85244, 85245, 85304, 85305, 85404, 85405 |
| **TBI-Related Conditions** | |
| Tinnitus^a^ | 38830, 38831, 38832, H9311, H9312, H9313, H9319 |
| Significant Hearing Loss^e^ | 3882, 3892, 3897, 3898, 3899, 38801, 38802, 38812, 38900, 38901, 38902, 38903, 38904, 38905, 38906, 38908, 38910, 38911, 38912, 38913, 38914, 38915, 38916, 38917, 38918, 38920, 38921, 38922, H8000, H8001, H8002, H8003, H8010, H8011, H8012, H8013, H8020, H8021, H8022, H8023, H8080, H8081, H8082, H8083, H8090, H8091, H8092, H8093, H833X1, H833X2, H833X3, H833X9, H900, H9011, H9012, H902, H903, H9041, H9042, H905, H906, H9071, H9072, H908, H90A11, H90A12, H90A21, H90A22, H90A31, H90A32, H9101, H9102, H9103, H9109, H9110, H9111, H9112, H9113, H9120, H9121, H9122, H9123, H913, H918X1, H918X2, H918X3, H918X9, H9190, H9191, H9192, H9193, H93011, H93012, H93013, H93019, H93091, H93092, H93093, H93099, H9400, H9401, H9402, H9403, V412, V532, Z461 |
| Hearing Problems^b^ | 38810–12, 3882, 38830–32, 38840, 38842–45, 3885, 3889, 38900–06, 38910–22, 3988, 3989, H833X9, H918X9, H833X9, H9319, H93249, H93299, H93239, H93219, H93299, H902, H9011, H9012, H900, H905, H903, H9041, H9042, H905, H9041, H9042, H905, H9041, H9042, H903, H908, H908, H9071, H9072, H906, H9123, H933X9, H9393, H918X9, H9190 |
| Dizziness/Vertigo^b^ | 38600, 38603, 38610–12, 38619, 3862 , 38630, 38635, 38640, 38642–43, 38650, 38653, 38658, 3869, 7804, H8109, H8113, H8123, H81319, H81399, H8149, H8193, H8309, H8319, H832X9, H8393, R42, H47619, H479, H4900, H4910, H4920, H4940, H49889, H5000, H50011, H50012, H5005, H5010, H50111, H50112, H5015, H5017, H5021, H5022, H5030, H50311, H50312, H5032, H50331, H50332, H5034, H5040, H50411, H50412, H5042, H5043, H5050, H5051, H5052, H5053, H5055, H5060, H5500, H5501, H5502, H5509, H5581, H5589, H5713, H578, H579 |
| Chronic Fatigue Syndrome^e^ | 278071, R5382 |
| Fatigue^c^ | 78071, 78072, 78079 |
| Sleep Apnea^e^ | 32720, 32721, 32722, 32723, 32724, 32725, 32726, 32727, 32729, 78050, 78051, 78053, 78057, G4730, G4731, G4733, G4734, G4735, G4736, G4737, G4739 |
| Sleep Disorders and Symptoms^b^ | 30740–49, 32700–02, 32709–15, 32719–24, 32726–27, 32729, 32730–31, 32733, 32735–36, 32739, 32740–44, 32749, 32751, 32753, 32759, 3278, 34700–01, 34710, 78050–59, F5101, F5102, F5103, F5104, F5105, F5109, F5111, F5112, F5113, F5119, F513, F518, F519, G4700, G4701, G4709, G4710, G4711, G4712, G4713, G4714, G4719, G4720, G4721, G4723, G4725, G4726, G4729, G4730, G4731, G4732, G4733, G4734, G4736, G4737, G4739, G47411, G47419, G47429, G4750, G4751, G4752, G4753, G4754, G4759, G4761, G4763, G4769, G478, G479 |
| Sleep Disruption Movement Disorders^d^ | 32751, 32752, 32753, 32759, 33394, 78058, G4761, G4762, G4763, G4769, G2581, F518 |
| Gait and Coordination Problems^b^ | 7812–3, R260, R261, R2689, R269, R270, R278, R279 |
| Skin Sensation Disturbances^b^ | 7820, R200, R201, R202, R203, R208, R209 |
| Vision Problems^b^ | 37775, 3779, 37800–01, 37805, 37810–11, 37815, 37817, 37820–24, 37830–35, 37840–43, 37845, 37850–55, 37860, 37950–52, 37954, 37956–58, 3798, 37990–93,  37775, 37790, 37800, 37801, 37805, 37810, 37811, 37815, 37817, 37820, 37821, 37822, 37823, 37824, 37830, 37831, 37832, 37833, 37834, 37835, 37840, 37841, 37842, 37843, 37845, 37850, 37851, 37852, 37853, 37854, 37855, 37860, 37950, 37951, 37952, 37954, 37956, 37957, 37958, 37980, 37990, 37991, 37992, 37993, H47619, H479, H4900, H4910, H4920, H4940, H49889, H5000, H50011, H50012, H5005, H5010, H50111, H50112, H5015, H5017, H5021, H5021, H5022, H5030, H50311, H50312, H5032, H50331, H50332, H5034, H5040, H50411, H50412, H5042, H5043, H5050, H5051, H5052, H5053, H5055, H5060, H5500, H5501, H5502, H5509, H5581, H5589, H5713, H578, H579 |
| Headache^b^ | 33910–12, 33920–22, 3393, 33941–44, 33982–85, 33989, 33900–03, 34600–03, 34610–13, 34620–21, 34630, 34640, 34651, 34670–73, 34680–82, 34690–93, 7840,  G43001, G43009, G43011, G43019, G43101, G43109, G43111, G43119, G43409, G43519, G43701, G43709, G43711, G43719, G43801, G43809, G43819, G43819, G43829, G43901, G43909, G43911, G43919, G43A1, G43B1, G43C1, G43D1, G44009, G44019, G44029, G44039, G441, G44209, G44219, G44221, G44229, G44309, G44319, G44329, G4441, G4451, G4452, G4453, G4459, G4482, G4483, G4484, G4485, G4489, R51 |
| Migraine Headaches^e^ | 3460, 3461, 3462, 3468, 3469, 33900, 33901, 33902, 33903, 33904, 33905, 33909, 34600, 34601, 34602, 34603, 34610, 34611, 34612, 34613, 34620, 34621, 34622, 34623, 34630, 34631, 34632, 34633, 34640, 34641, 34642, 34643, 34650, 34651, 34652, 34653, 34660, 34661, 34662, 34663, 34670, 34671, 34672, 34673, 34680, 34681, 34682, 34683, 34690, 34691, 34692, 34693, G43001, G43009, G43011, G43019, G43101, G43109, G43111, G43119, G43401, G43409, G43411, G43419, G43501, G43509, G43511, G43519, G43601, G43609, G43611, G43619, G43701, G43709, G43711, G43719, G43801, G43809, G43811, G43819, G43821, G43829, G43831, G43839, G43901, G43909, G43911, G43919, G43A0, G43A1, G43B0, G43B1, G43C0, G43C1, G43D0, G43D1, G44001, G44009, G44011, G44019, G44021, G44029, G44031, G44039, G44041, G44049, G44051, G44059, G44091, G44099 |
| Non-Headache Pain^b^ | 3078, 30789, 338–3384, 355, 3559, 356–3569, 3572, 3579, 5246, 710–73399, D481, E0842, E0942, E1042, E1142, E1342, F4541, F4542, G5700, G589, G600, G601, G603, G608, G609, G619, G890, G8911, G8912, G8918, G8921, G8922, G8928, G8929, G893, G894, M00019, M00029, M00039, M00049, M00059, M00069, M00079, M0008, M0009, M0010, M00119, M00129, M00139, M00149, M00159, M00169, M00179, M0018, M0019, M00219, M00229, M00239, M00249, M00259, M00269, M00279, M0028, M0029, M00819, M00829, M00839, M00849, M00859, M00869, M00879, M0088, M0089, M009, M01X0, M01X19, M01X29, M01X39, M01X49, M01X59, M01X69, M01X79, M01X8, M01X9, M0200, M0210, M02119, M02129, M02139, M02149, M02159, M02169, M02179, M0218, M0219, M0220, M0230, M02319, M02329, M02339, M02349, M02359, M02369, M02379, M0238, M0239, M029, M0500, M0510, M0530, M0560, M061, M064, M069, M0800, M083, M0840, M1120, M11219, M11229, M11239, M11249, M11259, M11269, M11279, M1128, M1129, M1180, M11819, M11829, M11839, M11849, M11859, M11869, M11879, M1188, M1189, M119, M1200, M1210, M12119, M12129, M12139, M12149, M12159, M12169, M12179, M1218, M1219, M1220, M12219, M12229, M12239, M12249, M12259, M12269, M12279, M1228, M1229, M1230, M12319, M12329, M12339, M12349, M12359, M12369, M12379, M1238, M1239, M1240, M12419, M12429, M12439, M12449, M12459, M12469, M12479, M1248, M1249, M1250, M12519, M12529, M12539, M12549, M12559, M12569, M12579, M1258, M1259, M1280, M12819, M12829, M12839, M12849, M12859, M12869, M12879, M1288, M1289, M129, M130, M1310, M13119, M13129, M13139, M13149, M13159, M13169, M13179, M1380, M13819, M13829, M13839, M13849, M13859, M13869, M13879, M1388, M1389, M1460, M1480, M150, M151, M152, M153, M158, M159, M1610, M167, M169, M1710, M175, M179, M189, M19019, M19029, M19039, M19049, M19079, M19219, M19229, M19239, M19249, M19279, M1990, M1991, M1993, M2010, M2240, M23009, M23202, M23205, M23219, M23229, M23239, M23249, M23259, M23269, M23305, M23319, M23329, M23339, M23349, M23359, M23369, M2340, M2350, M238X9, M2390, M2400, M24019, M24029, M24039, M24049, M24059, M24073, M24076, M2408, M2410, M24119, M24129, M24139, M24149, M24159, M24173, M24176, M2420, M2430, M24319, M24329, M24339, M24349, M24359, M24369, M24373, M24376, M2440, M24419, M24429, M24439, M24443, M24446, M24459, M24469, M24473, M24476, M2450, M24519, M24529, M24539, M24549, M24559, M24569, M24573, M24576, M2460, M24619, M24629, M24639, M24649, M24659, M24669, M24673, M24676, M247, M2480, M24819, M24829, M24839, M24849, M24859, M24873, M24876, M249, M2500, M25019, M25029, M25039, M25049, M25059, M25069, M25073, M25076, M2508, M2510, M25119, M25129, M25139, M25149, M25159, M25169, M25173, M25176, M2518, M2540, M25419, M25429, M25439, M25449, M25459, M25469, M25473, M25476, M2548, M2550, M25519, M25529, M25539, M25559, M25569, M25579, M2560, M25619, M25629, M25639, M25649, M25659, M25669, M25673, M25676, M2570, M25729, M2580, M25819, M25829, M25839, M25849, M25859, M25869, M25879, M259, M2660, M2669, M3210, M3320, M3390, M340, M341, M349, M3500, M3501, M352, M355, M357, M358, M359, M362, M363, M364, M4200, M4210, M4327, M4328, M434, M435X9, M436, M438X9, M459, M4600, M461, M4620, M4630, M4640, M4645, M4647, M4680, M4690, M4710, M4712, M4714, M4716, M47812, M47814, M47817, M47819, M4800, M4802, M4804, M4806, M4808, M4810, M4820, M4830, M4840XA, M4841XA, M4842XA, M4843XA, M4844XA, M4845XA, M4846XA, M4847XA, M4848XA, M4850XA, M489, M4980, M5000, M5020, M5030, M5080, M5090, M5104, M5105, M5106, M5107, M5124, M5125, M5126, M5127, M5134, M5135, M5136, M5137, M5144, M5145, M5146, M5147, M5184, M5185, M5186, M5187, M519, M530, M531, M532X7, M532X8, M533, M5382, M539, M5402, M5408, M5410, M5412, M5413, M5414, M5415, M5416, M5417, M542, M5430, M545, M546, M5489, M549, M60009, M6010, M6020, M609, M6100, M6110, M6140, M6159, M619, M6200, M6210, M623, M6240, M6250, M6281, M6282, M62838, M6289, M629, M6500, M6520, M6530, M654, M6580, M65849, M65879, M659, M6610, M6618, M66239, M66249, M66259, M66269, M66339, M66349, M66369, M66829, M66879, M6688, M669, M6700, M6740, M67419, M67429, M67439, M67449, M67459, M67469, M67479, M6750, M6780, M6788, M6790, M70039, M7010, M7020, M7030, M7040, M7050, M7060, M7070, M7098, M7100, M7120, M7130, M7140, M7150, M7180, M719, M720, M721, M722, M724, M726, M729, M7500, M75100, M75110, M75120, M7520, M7530, M7540, M7550, M7580, M7610, M7620, M7640, M7650, M7660, M76829, M76899, M7700, M7710, M7720, M7730, M7740, M7750, M778, M779, M790, M791, M792, M793, M794, M795, M79609, M79643, M79646, M797, M7981, M7989, M799, M79A19, M79A29, M79A3, M79A9, M8008XA, M810, M818, M8430XA, M84319A, M84329A, M84339A, M84343A, M84350A, M84353A, M84359A, M84369A, M84373A, M84376A, M84379A, M8438XA, M8440XA, M84419A, M84429A, M84439A, M84453A, M84459A, M84469A, M84479A, M8448XA, M8468XA, M8500, M852, M8530, M8540, M8550, M8560, M8610, M86119, M86129, M86139, M86149, M86159, M86169, M86179, M8618, M8619, M8620, M86219, M86229, M86239, M86249, M86259, M86269, M86279, M8628, M8629, M8660, M86619, M86629, M86639, M86642, M86659, M86669, M86679, M8668, M8669, M869, M8700, M87029, M87059, M87076, M8708, M889, M8900, M89129, M89139, M89159, M89169, M8918, M8930, M8940, M8960, M89619, M89629, M89639, M89649, M89659, M89669, M89679, M8968, M8969, M8970, M898X9, M899, M9060, M9080, M90819, M90829, M90839, M90849, M90859, M90869, M90879, M9088, M9089, M9180, M9230, M9240, M9250, M9260, M9270, M928, M93003, M931, M9320, M9380, M9390, M940, M9420, M948X9, M949, M961, R252, R262, R294, R29898, R52, S0291XK, S0292XK, S12000K, S12001K, S12100K, S12101K, S12200K, S12201K, S12300K, S12301K, S12400K, S12401K, S12500K, S12501K, S12600K, S12601K, S229XXK, S329XXK, S42009K, S42009P, S42209K, S42209P, S4290XK, S4290XP, S5290XK, S5290XM, S5290XN, S5290XP, S5290XQ, S5290XR, S6290XK, S6290XP, S7290XK, S7290XM, S7290XN, S7290XP, S7290XQ, S7290XR, S82009P, S82009Q, S82009R, S8290XK, S8290XM, S8290XN, S8290XP, S8290XQ, S8290XR, S92909K, S92909P, S92919K |
| Syncope and Collapse^b^ | 7802, R55 |
| Altered Mental Status^b^ | 78002, 78097, R404, R4182 |
| Cognitive Problems^b^ | 79951–55, 79959, 78093, 33183, R412, R413, R41840, R41841, R41842, R41843, R41844, R4189 |
| Communication Disorders^b^ | 7843, 78451, 78459–61, 78469, R471, R4702, R4781, R4789, R489, R480, R481, R482, R488, R4701 |
| Delirium/Dementia^b^ | 2930–1, 2940, 2948–9, 3010, 3102, 31089, 3109, F0789, F05, F04, F060, F068, F0781, F09 |
| Drug Abuse/Dependence^b^ | 30400–03, 30410–13, 30420–23, 30430–33, 30440–43, 30450, 30453, 30460–63, 30470, 30471–73, 30480–83, 30490–93, 30520–23, 30530, 30532–33, 30540–43, 30550–53, 30560–63, 30570–73, 30580–81, 30590–93, F1110, F1120, F1121, F1210, F1220, F1221, F1290, F1310, F1320, F1321, F1410, F1420, F1421, F1510, F1520, F1521, F1610, F1620, F1621, F1810, F1910, F1920, F1921 |
| Memory Loss^d^ | 411, R412, R413, 78093, 294 |
| Post-Concussive Syndrome^c^ | 3102 |
| **Mental Health Diagnoses** | |
| Acute Stress Disorder^b^ | 3080–4, 3089, F430, R457 |
| ADD/ADHD^b^ | 31400–01, 3148–9, F900, F901, F902, F908, F909 |
| Adjustment Disorders^a^ | 309, 3099, 3090, 3091, 30924, 30928, 3093, 3094, 30929, 3098, 30982, 30983, 30989, 3092, 30921, 30922, 30923, F342, F3420, F3421, F3422, F3423, F3424, F4325, F4329, F438, F439 |
| Anxiety Disorders^a^ | 3002, 30021, 30022, 30023, 30029, 30020, 3000, 30001, 30002, 30009, 30000, 3003, F40, F400, F4000, F4001, F4002, F401, F4010, F4011, F402, F4021, F40210, F40218, F4022, F40220, F40228, F4023, F40230, F40231, F40232, F40233, F4024, F40240, F40241, F40242, F40243, F40248, F4029, F40290, F40291, F40298, F408, F409, F41, F410, F411, F413, F418, F419, F42, F422, F423, F424, F428, F429 |
| Manic-Depressive Disorder^e^ | 2967, 29600, 29601, 29602, 29603, 29604, 29605, 29606, 29610, 29611, 29612, 29613, 29614, 29615, 29616, 29640, 29641, 29642, 29643, 29644, 29645, 29646, 29650, 29651, 29652, 29653, 29654, 29655, 29656, 29660, 29661, 29662, 29663, 29664, 29665, 29666, 29680, 29681, 29689, 29690, 29699, F3010, F3011, F3012, F3013, F302, F303, F304, F308, F309, F310, F3110, F3111, F3112, F3113, F312, F3130, F3131, F3132, F314, F315, F3160, F3161, F3162, F3163, F3164, F3170, F3171, F3172, F3173, F3174, F3175, F3176, F3177, F3178, F3181, F3189, F319 |
| Bipolar Disorders^a^ | 2960, 2961, 29600, 29610, 29601, 29611, 29602, 29612, 29603, 29613, 29604, 29614, F30, F3010, F3011, F3012, F3013, F302, 29605, 29615, 29606, 29616, 29681, 2964, 29640, 29641, 29642, 29643, 29644, 2965, 29650, 29651, 29652, 29653, 29654, 2966, 29660, F303, F304, F308, F309, F31, F310, F311, F3110, F3111, F3112, F3113, F312, F313, F3130, F3131, F3132, F314, F315, F316, F3160, 29661, 29662, 29663, 29664, 2967, 29645, 29646, 29655, 29656, 29665, 29666, 2968, 29682, 29689, 29640, 29680, 30113, F3161, F3162, F3163, F3164, F317, F3170, F3171, F3172, F3173, F3174, F3175, F3176, F3177, F3178, F318, F3181, F3189, F319, F340 |
| Depressive Disorders^a^ | 2962, 29621, 29622, 29623, 29624, 29625, 29626, 29620, 311, 2963, 29631, 29632, 29633, 29634, 29630, 29635, 29636, 29699, 29630, 3004, 29699, 2969, 29690, F32, F320, F321, F322, F323, F324, F325, F328, F3281, F3289, F329, F33, F330, F331, F332, F333, F334, F3340, F3341, F3342, F338, F339, F34, F341, F348, F3481, F3489, F349, F39 |
| Personality Disorders^a^ | 30122, 30110, 30112, 301, 3010, 3011, 3012, 30120, 3017, 30183, 3013, 3015, 30150, 30159, 3014, 30182, 3016, 3018, 30181, 30111, 30184, 30189, 3019, F21, F60, F600, F601, F602, F603, F604, F605, F606, F607, F608, F6081, F6089, F609 |
| PTSD^a^ | 30981, F431, F4310, F4311, F4312 |
| Schizophrenia^a^ | F20, F200, F201, F202, F203, F205, F208, F2081, F2089, F209, F25, F250, F251, F258, F259, 295, 2953, 29530, 29531, 29532, 29533, 29534, 29535, 2951, 29510, 29511, 29512, 29513, 29514, 29515, 2952, 29520, 29521, 29522, 29523, 29524, 29525, 29590, 2956, 29560, 29561, 29562, 29563, 29564, 29565, 2954, 29540, 29541, 29542, 29543, 29544, 29545, 2950, 29500, 29501, 29502, 29503, 29504, 29505, 2958, 29580, 29581, 29582, 29583, 29584, 29585, 2955, 29550, 29551, 29552, 29553, 29554, 29555, 2959, 29590, 29591, 29592, 29593, 29594, 29595, 2957, 29570, 29571, 29572, 29573, 29574, 29575 |
| ^a^Armed Forces Health Surveillance Division; all conditions required 1 inpatient or 2 outpatient visits within 180 days except for schizophrenia was required 1 inpatient or 4 outpatient visits without a time limit in accordance with these criteria  ^b^(Farmer et al., 2017); sensitive criteria.  ^c^(Belding, Khokhar, Englert, et al., 2021); sensitive criteria.  ^d^(Belding et al., 2024b); criterion of two inpatient or outpatient visits within one year.  ^e^(Carey, Harbertson, Sharifian, Boyko, Rull, et al., 2024); sensitive criteria. | |

| **Supplemental Table 2.** Demographic and Military Characteristics among Veterans enrolled in the Millennium Cohort Study who were enrolled in the VHA for 2 or more years. | | | | | | | | | | |
| --- | --- | --- | --- | --- | --- | --- | --- | --- | --- | --- |
|  | **All**  **n=51,541** | | **Low-level blast (LLB)** | | | | **High-level blast (HLB)** | | | |
|  |  |  | **No**  **n=43,368** | | **Yes**  **n=8,173** | | **No**  **n=46,932** | | **Yes**  **n=4,509** | |
|  | **n** | **(%)** | **n** | **(%)** | **n** | **(%)** | **n** | **(%)** | **n** | **(%)** |
| **Panel (Enrollment years)** |  |  |  |  |  |  |  |  |  |  |
| 1 (2001-2003) | 18,654 | (36.2) | 16,440 | (37.9) | 2,214 | (27.1) | 17,402 | (37.1) | 1,252 | (27.2) |
| 2 (2004-2006) | 6,267 | (12.2) | 5,230 | (12.1) | 1,037 | (12.7) | 5,592 | (11.9) | 675 | (14.6) |
| 3 (2007-2008) | 8,929 | (17.3) | 7,550 | (17.4) | 1,379 | (16.9) | 8,007 | (17.1) | 922 | (20.0) |
| 4 (2011-2013) | 17,691 | (34.3) | 14,148 | (32.6) | 3,543 | (43.4) | 15,931 | (33.9) | 1,760 | (38.2) |
| **Sex** |  |  |  |  |  |  |  |  |  |  |
| Male | 35,960 | (69.8) | 28,496 | (65.7) | 7,464 | (91.3) | 31,824 | (67.8) | 4,136 | (89.7) |
| Female | 15,581 | (30.2) | 14,872 | (34.3) | 709 | (8.7) | 15,108 | (32.2) | 473 | (10.3) |
| **Race and ethnicity** |  |  |  |  |  |  |  |  |  |  |
| American Indian | 814 | (1.6) | 655 | (1.5) | 159 | (1.9) | 735 | (1.6) | 79 | (1.7) |
| Asian or Pacific Islander | 2,160 | (4.2) | 1,881 | (4.3) | 279 | (3.4) | 2,020 | (4.3) | 140 | (3.0) |
| Black, non-Hispanic | 6,267 | (12.2) | 5,618 | (13.0) | 649 | (7.9) | 5,939 | (12.7) | 328 | (7.1) |
| White, non-Hispanic | 37,322 | (72.4) | 31,051 | (71.6) | 6,271 | (76.7) | 33,647 | (71.7) | 3,675 | (79.7) |
| Hispanic | 4,288 | (8.3) | 3,562 | (8.2) | 726 | (8.9) | 3,942 | (8.4) | 346 | (7.5) |
| Multiracial | 690 | (1.3) | 601 | (1.4) | 89 | (1.1) | 649 | (1.4) | 41 | (0.9) |
| **Birth year** |  |  |  |  |  |  |  |  |  |  |
| Before 1970 | 11,869 | (23.0) | 10,702 | 24.7 | 1,167 | 14.3 | 11,131 | (23.7) | 738 | (16.0) |
| 1970-1979 | 12,070 | (23.4) | 10,260 | 23.7 | 1,810 | 22.1 | 10,969 | (23.4) | 1,101 | (23.9) |
| After 1979 | 27,602 | (53.6) | 22,406 | 51.7 | 5,196 | 63.6 | 24,832 | (52.9) | 2,770 | (60.1) |
| **Education** |  |  |  |  |  |  |  |  |  |  |
| High School Diploma/ equivalent or less | 6,430 | (12.5) | 4,588 | (10.6) | 1,842 | (22.5) | 5,580 | (11.9) | 850 | (18.4) |
| Some college, no degree | 20,111 | (39.0) | 16,180 | (37.3) | 3,931 | (48.1) | 17,996 | (38.3) | 2,115 | (45.9) |
| Associates degree | 7,765 | (15.1) | 6,640 | (15.3) | 1,125 | (13.8) | 7,175 | (15.3) | 590 | (12.8) |
| Bachelors degree | 10,052 | (19.5) | 9,049 | (20.9) | 1,003 | (12.3) | 9,372 | (20.0) | 680 | (14.8) |
| Masters or higher | 7,183 | (13.9) | 6,911 | (15.9) | 272 | (3.3) | 6,809 | (14.5) | 374 | (8.1) |
| **Marital status** |  |  |  |  |  |  |  |  |  |  |
| Single, never married | 10,831 | (21.0) | 8,983 | (20.7) | 1,848 | (22.6) | 10,033 | (21.4) | 798 | (17.3) |
| Now married | 32,262 | (62.6) | 27,169 | (62.6) | 5,093 | (62.3) | 29,277 | (62.4) | 2,985 | (64.8) |
| No longer married | 8,448 | (16.4) | 7,216 | (16.6) | 1,232 | (15.1) | 7,622 | (16.2) | 826 | (17.9) |
| **Pay grade** |  |  |  |  |  |  |  |  |  |  |
| Enlisted | 42,168 | (81.8) | 34,147 | (78.7) | 8,021 | (98.1) | 38,115 | (81.2) | 4,053 | (87.9) |
| Officer | 9,373 | (18.2) | 9,221 | (21.3) | 152 | (1.9) | 8,817 | (18.8) | 556 | (12.1) |
| **Service branch** |  |  |  |  |  |  |  |  |  |  |
| Army | 21,587 | (41.9) | 16,768 | (38.7) | 4,819 | (59.0) | 18,269 | (38.9) | 3,318 | (72.0) |
| Navy/Coast Gard | 10,169 | (19.7) | 8,876 | (20.5) | 1,293 | (15.8) | 9,940 | (21.2) | 229 | (5.0) |
| Marine Corps | 6,185 | (12.0) | 4,801 | (11.1) | 1,384 | (16.9) | 5,418 | (11.5) | 767 | (16.6) |
| Air Force | 13,600 | (26.4) | 12,923 | (29.8) | 677 | (8.3) | 13,305 | (28.3) | 295 | (6.4) |
| **Days deployed**  (geometric mean, std dev) | 292.4 (1.4) | | 273.6 (1.5) | | 389.0 (3.5) | | 276.4 (1.5) | | 441.2 (4.6) | |
| **Deployment experience** |  |  |  |  |  |  |  |  |  |  |
| Not deployed | 17,627 | (34.2) | 15,948 | (36.8) | 1,679 | (20.5) | 17,233 | (36.7) | 394 | (8.5) |
| Deployed, without combat | 5,413 | (10.5) | 4,949 | (11.4) | 464 | (5.7) | 5,363 | (11.4) | 50 | (1.1) |
| Deployed, with combat | 28,501 | (55.3) | 22,471 | (51.8) | 6,030 | (73.8) | 24,336 | (51.9) | 4,165 | (90.4) |
| **High-level blast** |  |  |  |  |  |  |  |  |  |  |
| No | 46,932 | (91.1) | 40,810 | (94.1) | 6,122 | (74.9) | 46,932 | (100.0) | . | . |
| Yes | 4,609 | (8.9) | 2,558 | (5.9) | 2,051 | (25.1) | . | . | 4,609 | (100.0) |
| **Low-level blast** |  |  |  |  |  |  |  |  |  |  |
| No | 43,368 | (84.1) | 43,368 | (100) |  |  | 40,810 | (87.0) | 2,558 | (55.5) |
| Yes | 8,173 | (15.9) |  |  | 8,173 | (100) | 6,122 | (13.0) | 2,051 | (44.5) |
| **High-Risk LLB Occupations** |  |  |  |  |  |  |  |  |  |  |
| Armor and Amphibious, General | 391 | (6.1) |  |  | 391 | (6.1) | 277 | (6.0) | 114 | (6.5) |
| Artillery and Gunnery | 485 | (7.6) |  |  | 485 | (7.6) | 360 | (7.8) | 125 | (7.1) |
| Aviation Ordnance | 511 | (8.0) |  |  | 511 | (8.0) | 492 | (10.7) | 19 | (1.1) |
| Combat Engineering, General | 514 | (8.1) |  |  | 514 | (8.1) | 327 | (7.1) | 187 | (10.6) |
| Combat Operations Control, General | 807 | (12.6) |  |  | 807 | (12.6) | 561 | (12.1) | 246 | (14.0) |
| EOD/UDT | 193 | (3.0) |  |  | 193 | (3.0) | 143 | (3.1) | 50 | (2.8) |
| Expeditionary Medical Service | 270 | (4.2) |  |  | 270 | (4.2) | 226 | (4.9) | 44 | (2.5) |
| Infantry, General | 2419 | (37.9) |  |  | 2419 | (37.9) | 1522 | (32.9) | 897 | (50.9) |
| Infantry, Gun Crews, and Seamen | 173 | (2.7) |  |  | 173 | (2.7) | 173 | (3.7) | 0 | (.) |
| Military Training Instructor | 148 | (2.3) |  |  | 148 | (2.3) | 142 | (3.1) | 6 | (.3) |
| Missile Artillery, Operating | 304 | (4.8) |  |  | 304 | (4.8) | 277 | (6.0) | 27 | (1.5) |
| Rocket Artillery | 74 | (1.2) |  |  | 74 | (1.2) | 56 | (1.2) | 18 | (1.0) |
| Special Forces | 95 | (1.5) |  |  | 95 | (1.5) | 65 | (1.4) | 30 | (1.7) |
| *LLB – low-level blast; HLB – high-level blast; EOD/UDT – Explosive Ordnance Disposal/Underwater Demolition Team | | | | | | | | | | |

| **Supplemental Table 3.** Case Counts for Traumatic Brain Injury (TBI) and Mental Health Conditions of Interest | | | | | | |
| --- | --- | --- | --- | --- | --- | --- |
| **Condition** | **Enrolled in VHA care for 2 or more years, n=51,541** | | | **Diagnosed in VHA after 2013 survey*  n (%)** | | **Analytic sample** |
|  | **Total diagnosed* n (%)** | **Diagnosed in MDR before 2013 survey* n (%)** | **Diagnosed in VHA before 2013 survey*  n (%)** |  |  |  |
| **TBI Diagnoses** | | | | | | |
| Any TBI^a^ | 6,755 (13.1) | 3,678 (7.1) | 915 (1.8) | 1,607 | 3.2 | 50,626 |
| Mild TBI^a^ | 6,013 (11.7) | 3,412 (6.6) | 674 (1.3) | 1,078 | 2.1 | 50,867 |
| Moderate TBI^a^ | 2,207 (4.3) | 823 (1.6) | 340 (0.7) | 780 | 1.5 | 51,201 |
| Severe TBI^a^ | 100 (0.2) | 76 (0.1) | 8 (0.0) | 9 | 0 | 51,533 |
| Penetrating TBI^a^ | 98 (0.2) | 57 (0.1) | 19 (0.0) | 6 | 0 | 51,522 |
| **TBI-Related Conditions** | | | | | | |
| Tinnitus^a^ | 14,950 (29.0) | 3,753 (7.3) | 2,948 (5.7) | 7,171 | 14.8 | 48,593 |
| Significant Hearing Loss^e^ | 15,698 (30.5) | 6,889 (13.4) | 3,577 (6.9) | 6,324 | 13.2 | 47,963 |
| Hearing Problems^b^ | 17,790 (34.5) | 6,764 (13.1) | 4,073 (7.9) | 7,725 | 16.3 | 47,468 |
| Dizziness/Vertigo^b^ | 7,376 (14.3) | 1,226 (2.4) | 140 (0.3) | 3,241 | 6.3 | 51,401 |
| Chronic Fatigue Syndrome^e^ | 1,468 (2.8) | - | - | 874 | 1.7 | 51,541 |
| Fatigue^c^ | 18,588 (36.1) | 7,201 (14.0) | 603 (1.2) | 5,815 | 11.4 | 50,936 |
| Sleep Apnea^e^ | 22,695 (44.0) | 7,220 (14.0) | 2,317 (4.5) | 12,955 | 26.3 | 49,222 |
| Sleep Disorders and Symptoms^b^ | 31,560 (61.2) | 13,934 (27.0) | 3,731 (7.2) | 17,726 | 37.1 | 47,805 |
| Sleep Disruption Movement Disorders^d^ | 2290 (4.4) | 452 (0.9) | 87 (0.2) | 941 | 1.8 | 51,454 |
| Gait and Coordination Problems^b^ | 2515 (4.9) | - | - | 1,523 | 3 | 51,541 |
| Skin Sensation Disturbances^b^ | 7,731 (15.0) | - | - | 4,037 | 7.8 | 51,541 |
| Vision Problems^b^ | 5,707 (11.1) | 3,217 (6.2) | 328 (0.6) | 1,025 | 2 | 51,212 |
| Headache^b^ | 20,654 (40.1) | 7,357 (14.3) | 1,949 (3.8) | 11,716 | 23.6 | 49,592 |
| Migraine Headaches^e^ | 14,093 (27.3) | 6,097 (11.8) | 1,862 (3.6) | 7,755 | 15.6 | 49,679 |
| Non-Headache Pain^b^ | 47,186 (91.6) | 38,823 (75.3) | 9,582 (18.6) | 22,934 | 54.7 | 41,952 |
| Syncope and Collapse^b^ | 6,566 (12.7) | 3,235 (6.3) | 277 (0.5) | 1,641 | 3.2 | 51,264 |
| Altered Mental Status^b^ | 2542 (4.9) | 714 (1.4) | 62 (0.1) | 872 | 1.7 | 51,479 |
| Cognitive Problems^b^ | 6,636 (12.9) | 1,392 (2.7) | 451 (0.9) | 3,169 | 6.2 | 51,090 |
| Communication Disorders^b^ | 966 (1.9) | 128 (0.2) | 46 (0.1) | 391 | 0.8 | 51,495 |
| Delirium/Dementia^b^ | 752 (1.5) | 1 (0.0) | 3 (0.0) | 395 | 0.8 | 51,538 |
| Memory Loss^d^ | 895 (1.7) | 1 (0.0) | 298 (0.6) | 594 | 1.2 | 51,243 |
| Post-Concussive Syndrome^c^ | 1,661 (3.2) | 886 (1.7) | 181 (0.4) | 297 | 0.6 | 51,360 |
| **Mental Health Diagnoses** | | | | | | |
| Acute Stress Disorder^a^ | 2,910 (5.6) | 1898 (3.7) | 181 (0.4) | 338 | 0.7 | 51,360 |
| ADD/ADHD^b^ | 4,496 (8.7) | 1340 (2.6) | 236 (0.5) | 2,587 | 5 | 51,305 |
| Adjustment Disorders^a^ | 19,238 (37.3) | 9675 (18.8) | 3,593 (7.0) | 6,788 | 14.2 | 47,948 |
| Anxiety Disorders^a^ | 19,910 (38.6) | 5104 (9.9) | 1,839 (3.6) | 12,254 | 24.6 | 49,700 |
| Manic-Depressive Disorder^e^ | 4,849 (9.4) | 1182 (2.3) | 910 (1.8) | 2,555 | 5.1 | 50,631 |
| Bipolar Disorders^a^ | 2,777 (5.4) | 611 (1.2) | 402 (0.8) | 1,650 | 3.2 | 51,139 |
| Depressive Disorders^a^ | 22,225 (43.1) | 7545 (14.6) | 3,491 (6.8) | 13,679 | 28.5 | 48,047 |
| Personality Disorders^a^ | 2,280 (4.4) | 958 (1.9) | 242 (0.5) | 940 | 1.8 | 51,299 |
| PTSD^a^ | 15,787 (30.6) | 2728 (5.3) | 2,766 (5.4) | 11,331 | 23.2 | 48,775 |
| Schizophrenia^a^ | 368 (0.7) | 54 (0.1) | 75 (0.1) | 229 | 0.4 | 51,466 |
| ^*^Total diagnosed, diagnosed in MDR before 2013 survey, and diagnosed in VHA before 2013 survey are among the entire eligible sample (n=51,541); Numbers diagnosed in VHA after 2013 survey (outcomes of interest) were calculated among the condition specific analytic sample that excludes cases diagnosed in the VHA before the 2013 survey. Models adjusted for military (service branch, total days deployed, pay grade, deployment/combat status) and demographic factors (age, sex, race, ethnicity, education, marital status), enrollment panel, diagnosis of the condition in the MDR before the 2013 survey, and the interaction between HLB and LLB.  ^a^Armed Forces Health Surveillance Division; all conditions required 1 inpatient or 2 outpatient visits within 180 days except for schizophrenia which required 1 inpatient or 4 outpatient visits without a time limit in accordance with these criteria.  ^b^(Farmer et al., 2017); sensitive criteria.  ^c^(Belding, Khokhar, Englert, et al., 2021); sensitive criteria.  ^d^(Belding et al., 2024b); criterion of two inpatient or outpatient visits within one year.  ^e^(Carey, Harbertson, Sharifian, Boyko, Rull, et al., 2024); sensitive criteria. | | | | | | |

| **Supplemental Table 4:** Adjusted Prevalence Ratios between HLB and LLB on Subsequent Mental Health, Traumatic Brain Injury and Sensory/Symptomology Diagnosis in the VHA, among Millennium Cohort Study Participants Enrolled in the VHA for 2 or more years | | | | | | | | | |
| --- | --- | --- | --- | --- | --- | --- | --- | --- | --- |
| **Condition** | **Adjusted LLB** | | | **Adjusted HLB** | | | **HLB x LLB Interaction** | | |
|  | **PR** | **95% CI** | **p-value*** | **PR** | **95% CI** | **p-value*** | **PR** | **95% CI** | **p-value*** |
| **Traumatic Brain Injury Diagnoses** | | | | | | | | | |
| Any TBI^a^ | 1.16 | (1.00, 1.35) | 0.07 | 2.92 | (2.53, 3.38) | <.0001 | 0.86 | (0.70, 1.05) | 0.13 |
| Mild TBI^a^ | 1.14 | (0.94, 1.37) | 0.27 | 2.87 | (2.40, 3.44) | <.0001 | 0.93 | (0.72, 1.21) | 0.59 |
| Moderate TBI^a^ | 1.33 | (1.07, 1.66) | 0.03 | 3.76 | (3.06, 4.61) | <.0001 | 0.74 | (0.55, 1.00) | 0.07 |
| Severe TBI^a^ | - |  |  | - |  |  |  |  | - |
| Penetrating TBI^a^ | - |  |  | - |  |  |  |  | - |
| **TBI-Related Conditions** | | | | | | | | | |
| Tinnitus^a^ | 1.12 | (1.05, 1.19) | 0.001 | 1.37 | (1.26, 1.49) | <.0001 | 0.97 | (0.86, 1.10) | 0.68 |
| Significant Hearing Loss^e^ | 1.12 | (1.05, 1.20) | 0.002 | 1.48 | (1.36, 1.61) | <.0001 | 0.92 | (0.81, 1.05) | 0.23 |
| Hearing Problems^b^ | 1.13 | (1.07, 1.20) | 0.0001 | 1.35 | (1.25, 1.46) | <.0001 | 0.94 | (0.84, 1.05) | 0.27 |
| Dizziness/Vertigo^b^ | 1.01 | (0.91, 1.13) | 0.86 | 1.49 | (1.30, 1.70) | <.0001 | 0.98 | (0.79, 1.22) | 0.89 |
| Chronic Fatigue Syndrome^e^ | 0.96 | (0.76, 1.20) | 0.70 | 1.11 | (0.82, 1.51) | 0.56 | 0.79 | (0.46, 1.34) | 0.48 |
| Fatigue^c^ | 0.99 | (0.92, 1.08) | 0.88 | 1.33 | (1.21, 1.47) | <.0001 | 0.95 | (0.81, 1.11) | 0.55 |
| Sleep Apnea^e^ | 0.99 | (0.95, 1.03) | 0.60 | 1.20 | (1.14, 1.27) | <.0001 | 0.88 | (0.81, 0.96) | 0.005 |
| Sleep Disorders and Symptoms^b^ | 1.02 | (0.99, 1.06) | 0.19 | 1.21 | (1.16, 1.27) | <.0001 | 0. 88 | (0.82, 0.94) | 0.0001 |
| Sleep Disruption Movement Disorders^d^ | 1.34 | (1.10, 1.63) | 0.02 | 1.42 | (1.10, 1.83) | 0.03 | 0.96 | (0.65, 1.42) | 0.83 |
| Gait and Coordination Problems^b^ | 1.12 | (0.96, 1.31) | 0.21 | 1.67 | (1.38, 2.02) | <.0001 | 0.76 | (0.55, 1.05) | 0.12 |
| Skin Sensation Disturbances^b^ | 1.03 | (0.94, 1.14) | 0.50 | 1.42 | (1.26, 1.60) | <.0001 | 0.80 | (0.65, 0.97) | 0.03 |
| Vision Problems^b^ | 1.15 | (0.95, 1.40) | 0.20 | 1.63 | (1.28, 2.07) | 0.003 | 0.93 | (0.64, 1.35) | 0.69 |
| Headache^b^ | 1.11 | (1.05, 1.16) | <.0001 | 1.47 | (1.39, 1.55) | <.0001 | 0.93 | (0.85, 1.02) | 0.10 |
| Migraine Headaches^e^ | 1.11 | (1.04, 1.18) | 0.004 | 1.57 | (1.46, 1.69) | <.0001 | 1.03 | (0.92, 1.16) | 0.59 |
| Non-Headache Pain^b^ | 1.04 | (1.02, 1.07) | 0.002 | 1.18 | (1.14, 1.22) | <.0001 | 0.92 | (0.88, 0.97) | 0.002 |
| Syncope and Collapse^b^ | 1.11 | (0.96, 1.29) | 0.18 | 1.68 | (1.40, 2.01) | <.0001 | 0.70 | (0.52, 0.95) | 0.03 |
| Altered Mental Status^b^ | 1.06 | (0.87, 1.30) | 0.60 | 1.99 | (1.58, 2.52) | <.0001 | 0.77 | (0.53, 1.11) | 0.20 |
| Cognitive Problems^b^ | 1.19 | (1.08, 1.32) | 0.002 | 2.20 | (1.97, 2.45) | <.0001 | 0.82 | (0.70, 0.97) | 0.02 |
| Communication Disorders^b^ | 1.48 | (1.11, 1.99) | 0.04 | 1.79 | (1.22, 2.61) | 0.04 | 0.73 | (0.42, 1.29) | 0.31 |
| Delirium/Dementia^b^ | 1.20 | (0.87, 1.65) | 0.32 | 2.20 | (1.55, 3.11) | 0.004 | 1.32 | (0.79, 2.19) | 0.07 |
| Memory Loss^d^ | 1.45 | (1.14, 1.84) | 0.02 | 2.92 | (2.27, 3.76) | <.0001 | 0.80 | (0.56, 1.16) | 0.06 |
| Post-Concussive Syndrome^c^ | 1.11 | (0.76, 1.63) | 0.76 | 2.48 | (1.71, 3.61) | 0.002 | 1.19 | (0.69, 2.05) | 0.76 |
| **Mental Health Diagnoses** | | | | | | | | | |
| Acute Stress Disorder^a^ | 1.29 | (0.92, 1.80) | 0.24 | 2.09 | (1.39, 3.13) | 0.03 | 0.50 | (0.25, 1.00) | 0.13 |
| ADD/ADHD^b^ | 1.03 | (0.92, 1.16) | 0.57 | 1.03 | (0.92, 1.16) | 0.57 | 1.12 | (0.90, 1.40) | 0.34 |
| Adjustment Disorders^a^ | 1.28 | (1.20, 1.36) | <.0001 | 1.28 | (1.20, 1.36) | <.0001 | 0.75 | (0.67, 0.83) | <.0001 |
| Anxiety Disorders^a^ | 1.03 | (0.99, 1.08) | 0.19 | 1.03 | (0.99, 1.08) | 0.19 | 0.87 | (0.79, 0.96) | 0.01 |
| Bipolar Disorders^a^ | 1.07 | (0.92, 1.23) | 0.41 | 1.07 | (0.92, 1.23) | 0.41 | 0.65 | (0.48, 0.89) | 0.01 |
| Manic-Depressive Disorder^e^ | 1.01 | (0.90, 1.14) | 0.85 | 1.01 | (0.90, 1.14) | 0.85 | 0.84 | (0.66, 1.05) | 0.14 |
| Depressive Disorders^a^ | 1.05 | (1.01, 1.10) | 0.01 | 1.05 | (1.01, 1.10) | 0.01 | 0.86 | (0.79, 0.93) | 0.0002 |
| Personality Disorders^a^ | 1.13 | (0.92, 1.38) | 0.32 | 1.13 | (0.92, 1.38) | 0.32 | 1.18 | (0.76, 1.82) | 0.49 |
| PTSD^a^ | 1.20 | (1.15, 1.26) | <.0001 | 1.20 | (1.15, 1.26) | <.0001 | 0.78 | (0.73, 0.84) | <.0001 |
| Schizophrenia^a^ | 1.20 | (0.84, 1.73) | 0.40 | 1.20 | (0.84, 1.73) | 0.40 | 0.74 | (0.34, 1.63) | 0.49 |
| LLB – low-level blast, determined by occupational risk; HLB – high-level blast, self-reported. Models adjusted for military (service branch, total days deployed, pay grade, deployment/combat status) and demographic factors (age, sex, race, ethnicity, education, marital status), enrollment panel, diagnosis of the condition in the MDR before the 2013 survey, and the interaction between HLB and LLB.  *False discovery rate (FDR) adjusted p-values  ^a^Armed Forces Health Surveillance Division; all conditions required 1 inpatient or 2 outpatient visits within 180 days except for schizophrenia was required 1 inpatient or 4 outpatient visits without a time limit in accordance with these criteria.  ^b^Farmer et al, 2016; sensitive criteria.  ^c^Belding et al, 2021; sensitive criteria.  ^d^Belding et al, under review; criterion of two inpatient or outpatient visits within one year.  ^e^Carey et al., 2024; sensitive criteria. | | | | | | | | | |

| **Supplemental Table 5.** Adjusted Prevalence Ratios examining the combined effect between HLB and LLB exposure on Subsequent Mental Health, Traumatic Brain Injury and Sensory/Symptomology Diagnosis in the VHA. | | | | |
| --- | --- | --- | --- | --- |
| **Condition** | **Ref: Neither** | **LLB Only** | **HLB Only** | **HLB and LLB** |
| **TBI-Related Conditions** | | | | |
| Sleep Apnea^c^ | Ref | 0.99 (0.95, 1.03) | **1.20 (1.14, 1.27)** | 1.05 (0.98, 1.11) |
| Sleep Disorders and Symptoms^b^ | Ref | 1.02 (0.99, 1.06) | **1.21 (1.16, 1.27)** | **1.09 (1.04, 1.14)** |
| Skin Sensation Disturbances^b^ | Ref | 1.03 (0.94, 1.14) | **1.42 (1.26, 1.60)** | **1.17 (1.01, 1.35)** |
| Non-Headache Pain^b^ | Ref | **1.04 (1.02, 1.07)** | **1.18 (1.14, 1.22)** | **1.13 (1.09, 1.17)** |
| Syncope and Collapse^b^ | Ref | 1.11 (0.96, 1.29) | **1.68 (1.40, 2.01)** | **1.31 (1.05, 1.64)** |
| Cognitive Problems^b^ | Ref | **1.19 (1.08, 1.32)** | **2.20 (1.97, 2.45)** | **2.15 (1.92, 2.42)** |
| **Mental Health Diagnoses** | | | | |
| Adjustment Disorders^a^ | Ref | **1.28 (1.20, 1.36)** | **1.82 (1.70, 1.95)** | **1.74 (1.61, 1.87)** |
| Anxiety Disorders^a^ | Ref | 1.03 (0.99, 1.08) | **1.22 (1.14, 1.29)** | **1.09 (1.02, 1.17)** |
| Bipolar Disorders^a^ | Ref | 1.07 (0.92, 1.23) | **1.53 (1.28, 1.85)** | 1.07 (0.84, 1.36) |
| Depressive Disorders^a^ | Ref | **1.05 (1.01, 1.10)** | **1.32 (1.26, 1.40)** | **1.19 (1.12, 1.27)** |
| PTSD^a^ | Ref | **1.20 (1.15, 1.26)** | **1.64 (1.56, 1.72)** | **1.55 (1.47, 1.63)** |
| LLB – low-level blast, determined by occupational risk; HLB – high-level blast, self-reported. Bold typeface indicates p < .05.  ^a^Armed Forces Health Surveillance Division; all conditions required 1 inpatient or 2 outpatient visits within 180 days.  ^b^Farmer et al, 2016; sensitive criteria. | | | | |
